# Extracellular Vesicles miRNome Profiling Reveals miRNAs Engagement in Dysfunctional Lipid Metabolism, Chronic Inflammation and Liver Damage in Subjects With Metabolic Dysfunction‐Associated Steatotic Liver Disease

**DOI:** 10.1111/apt.70150

**Published:** 2025-04-10

**Authors:** Gian Paolo Caviglia, Elisabetta Casalone, Chiara Rosso, Serena Aneli, Alessandra Allione, Fabrizia Carli, Cristina Grange, Angelo Armandi, Chiara Catalano, Giovanni Birolo, Beatrice Foglia, Davide Giuseppe Ribaldone, Amalia Gastaldelli, Giuseppe Matullo, Elisabetta Bugianesi

**Affiliations:** ^1^ Division of Gastroenterology and Hepatology, Department of Medical Sciences University of Turin Turin Italy; ^2^ Unit of Genomic, Department of Medical Sciences University of Turin Turin Italy; ^3^ Cardiometabolic Risk Unit Institute of Clinical Physiology, CNR Pisa Italy; ^4^ Division of Internal Medicine, Department of Medical Sciences University of Turin Turin Italy; ^5^ Metabolic Liver Disease Research Program, I. Department of Medicine University Medical Center of the Johannes Gutenberg‐University Mainz Germany; ^6^ Department of Clinical and Biological Sciences University of Turin Turin Italy

**Keywords:** FFA, insulin resistance, MASLD, miRNA, steatohepatitis

## Abstract

**Background and Aims:**

MicroRNAs (miRNAs) are short non‐coding oligonucleotides involved in the post‐transcriptional regulation of gene expression. We investigated the association between the miRNome profile of circulating extracellular vesicles (EVs) and metabolic derangements, circulating and hepatic pro‐inflammatory cytokines, and liver damage across the histological spectrum of metabolic dysfunction‐associated steatotic liver disease (MASLD).

**Methods:**

EV miRNAs expression was determined by NGS (NextSeq550, Illumina Inc) in 228 biopsy‐proven MASLD patients. In vivo metabolic studies were performed in a subgroup of 54 patients by tracer infusion ([6,6‐^2^H_2_]glucose and [^2^H_5_]glycerol) to assess glucose and lipid fluxes and insulin resistance (IR) in the adipose tissue.

**Results:**

Seven miRNAs (miR‐27b‐3p, miR‐30a‐5p, miR‐122‐5p, miR‐375‐3p, miR‐103a‐3p, let‐7d‐5p, and let‐7f‐5p) were differentially expressed according to the diagnosis of steatohepatitis and the presence of significant fibrosis (*F* ≥ 2), thus marking subjects with ‘at‐risk MASH’. In the metabolic studies, the above‐reported miRNAs had the strongest associations with lipid metabolism: miR‐122‐5p and miR‐375‐3p levels directly correlated with circulating free fatty acids (FFAs) and adipose tissue (AT)‐IR, while let‐7d‐5p and let‐7f‐5p inversely correlated with lipolysis, FFAs, and progressively decreased according to AT‐IR severity. In addition, let‐7d‐5p and let‐7f‐5p inversely correlated with the circulating and hepatic expression of pro‐inflammatory cytokines, which increased by increasing degrees of AT‐IR.

**Conclusions:**

Our results suggest an intertwined connection between miR‐122‐5p, miR‐375‐3p, and the let‐7 family in modulating lipid derangements and inflammatory pathways in patients with ‘at‐risk MASH’, paving the basis for further studies aiming at investigating their potential therapeutic value.

## Graphical abstract

This study explores the association between the miRNome profile of circulating extracellular vesicles and metabolic dysfunction, liver inflammation, and damage in patients with metabolic MASLD, identifying key miRNAs (miR‐122‐5p, miR‐375‐3p, let‐7d‐5p, and let‐7f‐5p) that modulate lipid metabolism and inflammatory pathways in ‘at‐risk MASH’ patients.

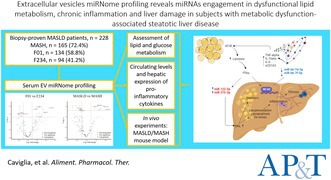

**Abbreviations**
γGT: gamma‐glutamyl transferase
AdipoR2: adiponectin receptor 2
ALT: alanine aminotransferase
AMPK: AMP‐activated protein kinase
AST: aspartate aminotransferase
AT‐IR: adipose tissue insulin resistance
BMI: body mass index
CDAA: choline‐deficient L‐aminoacid–defined diet
CI: confidence interval
CSAA: choline‐sufficient L‐amino acid–defined diet
DNL: de novo lipogenesis
EGP: endogenous glucose production
EVs: extracellular vesicles
FC: fold change
FE: fold expression
FFAs: free fatty acids
GC–MS: gas chromatography–mass spectrometry
GMC‐SF: granulocyte‐macrophage colony‐stimulating factor
HCC: hepatocellular carcinoma
HDL: high‐density lipoprotein
Hep‐IR: hepatic insulin resistance
HOMA‐IR: homeostasis model assessment of insulin resistance
IFNγ: interferon gamma
IL: interleukin
IQR: interquartile range
KEGG: Kyoto Encyclopedia of Genes and Genomes
LKB1: liver kinase B1
MASH: metabolic‐dysfunction associated steatohepatitis
MASLD: metabolic dysfunction‐associated steatotic liver disease
MCP‐1: monocyte chemoattractant protein‐1
MetS: metabolic syndrome
miRNAs: microRNAs
MUFAs: monounsaturated fatty acids
*n*
: number
NF‐kB: nuclear factor kappa B
PPAR: peroxisome proliferator‐activated receptor
PUFAs: α, polyunsaturated fatty acids
qPCR: quantitative polymerase chain reaction
Ra: rate of appearance
SFA: saturated fatty acids
T2DM: type 2 diabetes mellitus
TNFα: tumour necrosis factor alpha
TTR: tracer/trace ratios

## 1 Introduction

Metabolic dysfunction‐associated steatotic liver disease (MASLD) is a chronic clinical condition characterised by metabolically driven hepatic fat accumulation ranging from simple steatosis to steatohepatitis (MASH) [1], the latter characterised by inflammation, hepatocyte ballooning, and varying degrees of fibrosis, marking it as a high‐risk phenotype for adverse outcomes [2]. It is estimated that 30% of the total adult population is affected by MASLD, and the epidemiological burden of the disease is steadily growing in parallel to the pandemic of obesity and type 2 diabetes mellitus (T2DM) [3, 4, 5].

Although the pathogenesis of MASLD is multifactorial, insulin resistance (IR) is considered the major determinant for the onset and progression of the disease [6]. Specifically, IR in the adipose tissue determines a massive flux of (free fatty acids) that from the adipocytes reaches the liver; the excess of FFA in the hepatocyte contributes to the development of hepatic steatosis that in turn enhances lipotoxicity, lipoapoptosis, and hepatic fibrogenesis [7].

MicroRNAs (miRNAs) are short non‐coding RNAs responsible for post‐transcriptional regulation of gene expression. MiRNAs have cell‐specific expression profiles and play crucial roles in maintaining metabolic processes, including lipid and glucose metabolism [8]. Circulating miRNAs, particularly those packaged in extracellular vesicles (EVs), offer increased stability and specificity as biomarkers, reflecting the pathological state of their tissue of origin. However, the specific roles of EV‐derived miRNAs in MASLD progression, from steatosis to MASH and fibrosis, remain poorly defined [9].

In the present study, we performed a comprehensive miRNA profiling in circulating EVs from a cohort of biopsy‐proven MASLD patients. We hypothesised that distinct miRNAs are associated with the key pathological features of MASLD (i.e., steatosis, MASH, and fibrosis) and are involved in specific metabolic and inflammatory pathways. By correlating EV‐derived miRNA profiles with histological damage, metabolic fluxes, and cytokine expression, this study seeks to identify miRNAs relevant to the deranged metabolic pathways in MASLD.

## 2 Patients and Methods

### 2.1 Study Population

This cross‐sectional study includes patients with biopsy‐proven MASLD consecutively enrolled at the Liver Unit of the Department of Medical Sciences, University of Torino, Turin, Italy between June 2010 and May 2020. Inclusion criteria were age ≥ 18 years, a diagnosis of MASLD confirmed by liver biopsy, and the availability of a frozen serum sample collected at the time of liver biopsy. Other aetiologies of liver disease, including viral, autoimmune, cholestatic, genetic, alcoholic, and drug‐induced, were excluded. At the time of liver biopsy, a complete medical history and physical examination were undertaken, and anthropometric data was collected. Alcohol consumption was assessed by direct interview of the patient and a close relative; a negative history of alcohol abuse was considered for a weekly ethanol consumption < 140 g in women and < 210 g in men [10]. All patients underwent serological screening for hepatitis B surface antigen, antibodies to hepatitis B core antigen, and antibodies to hepatitis C virus. Diagnosis of metabolic syndrome (MetS) was defined according to the presence of at least three of the following criteria: waist circumference > 94 cm (men) or > 80 cm (women), blood pressure > 130/85 mmHg, fasting triglycerides level > 150 mg/dL, fasting high‐density lipoprotein (HDL) cholesterol ≤ 40 mg/dL (men) or ≤ 50 mg/dL (women), and fasting glucose > 100 mg/dL [11]. The homeostasis model assessment of insulin resistance (HOMA‐IR) was calculated according to the formula: [(fasting plasma insulin in mU/L) × (fasting plasma glucose in mmol/L)/22.5] [12].

All patients signed a written informed consent for the collection of personal data and for the use of blood samples for research purposes. This study was conducted according to the guidelines of the Declaration of Helsinki as revised in 2008 and approved by the Institutional Ethics Committee of A.O.U. Città della Salute e della Scienza di Torino (CEI/522, 17 November 2015).

#### 2.1.1 Isolation of Serum Extracellular Vesicles and RNA Extraction

EVs were isolated from 200 μL of serum previously collected and stored at −80°C, using ExoQuick precipitation solution (System Biosciences, USA) according to the manufacturer's instructions [13]. The miRNeasy serum/plasma kit (Qiagen, Germany) was used to extract total RNA with the QIAcube extractor (Qiagen, Germany) according to the manufacturer's instructions. RNA concentration was determined for all samples with Qubit 2.0 Fluorometer with miRNA assay kit (ThermoFisher, USA).

### 2.2 Library Preparation and Next‐Generation Sequencing

Small RNA libraries were constructed using NEBNext Multiplex Small RNA Library Prep set for Illumina (New England Biolabs Inc., USA). The cDNA libraries were purified with the Qiagen PCR Purification kit following the modifications indicated by NEBNext Multiplex Small RNA Library Prep instructions, and then 24 cDNA purified barcoded samples were pooled together. Each pool was finally enriched for microRNAs in a 6% PolyAcrylamide Gel (ThermoFisher, USA), fragments with an insert of 150 nucleotides were cut out and purified with Qiagen Gel Extraction MiniElute kit (Qiagen, Germany). Single‐end sequencing (75 nt) was performed on NextSeq550 (Illumina, USA).

### 2.3 Additional Methods

Additional methods were reported and described in the Supporting Information: Methods section. Briefly, we adopted nanoparticle tracking analysis to measure EV concentration, while super‐resolution microscopy to identify EV surface markers. Glucose and lipid metabolism were assessed via stable isotope infusions and gas chromatography–mass spectrometry (GC–MS). Free fatty acids were quantified by enzymatic assays and GC–MS. Gene expression of inflammatory markers was analysed through RT‐qPCR. Cytokines and related biomarkers were measured using ELISA and multiplex immunoassays. MASLD severity was histologically assessed; ‘at risk MASH’ was defined as NAFLD Activity Score (NAS) ≥ 4 and fibrosis stage ≥ 2. MiRNA sequencing involved differential expression analysis, target enrichment, and pathway mapping using ShinyGO and PathfindR.

### 2.4 Animal Experimentation

Eight‐week‐old male *wild‐type* mice were fed either a choline‐deficient L‐amino acid–defined (CDAA, *n* = 8) diet (Laboratorio Dottori Piccioni, Gessate, Italy) or the corresponding choline‐sufficient L‐amino acid–defined (CSAA, *n* = 6) diet for 24 weeks. The characteristics of CDAA‐fed mouse livers recapitulate the MASLD/MASH conditions [14]. These mice showed: (a) significant liver steatosis, with high levels of hepatic triglycerides and histological evidence of lipid accumulation; (b) evident fibrosis as shown by Sirius Red staining and increased expression of fibrogenic genes (collagen 1A1, α‐SMA, TGF‐β1); (c) lobular inflammation and infiltration of F4/80+ macrophages. The experiments complied with national ethical guidelines for animal experimentation, and the experimental protocols were approved by the Italian Ministry of Health.

### 2.5 Statistical Analysis

Methods concerning data analysis from miRNA sequencing are reported in Supporting Information: Materials and Methods. Continuous variables were reported as median and interquartile range (IQR) while categorical variables as absolute number (*n*) and percentage (%). Data normality was checked by the D'Agostino‐Pearson test. Mann–Whitney test and Kruskal‐Wallis test were used to compare continuous variables between two or more groups, respectively. Fisher's exact test or chi‐squared (*χ* ^2^) test were used to compare dichotomous data between groups. The correlation between continuous variables was calculated using Spearman's rank correlation. Correlation coefficients (*r*) were reported with the corresponding 95% confidence intervals (CI). Statistical analyses were performed using MedCalc software v. 18.9.1 (MedCalc Software Ltd., Ostend, Belgium) and R software (https://www.R‐project.org/). For all the analyses, a nominal *p* < 0.05 was considered statistically significant.

## 3 Results

### 3.1 Study Cohort Characteristics

A total of 228 patients with biopsy‐proven MASLD were included in the present study. The demographic, clinical, and biochemical characteristics are reported in Table 1. Median age was 48.5 (37.5–58.0) years and most patients were female (64.0%); 38.6% of patients were obese and 33.3% had T2DM. Overall, 40.4% of patients met the criteria for the diagnosis of MetS; the most common feature of MetS was represented by central obesity (*n* = 175, 76.8%), followed by low HDL cholesterol (*n* = 96, 42.1%), hypertension (*n* = 91, 39.9%), hyperglycemia (*n* = 82, 36.0%), and hypertriglyceridemia (*n* = 73, 32.0%). A histological diagnosis of steatohepatitis was made in 165 subjects (72.4%), according to the concomitant presence of liver steatosis > 5%, lobular inflammation, and hepatocyte ballooning. A significant liver fibrosis (defined as *F* ≥ 2) was observed in 94 (41.2%) patients; 53 (23.3%) patients were ‘at risk MASH’.

**TABLE 1:** Characteristics of the whole study cohort, and according to the diagnosis of ‘at‐risk MASH’.

Variables	Total, *n* = 228	‘At‐risk MASH’, *n* = 53 (23.2%)	Other, *n* = 175 (76.8%)	*p*
Age (years), median (IQR)	48.5 (37.5–58.0)	53.0 (37.5–61.0)	47.0 (37.3–57.0)	0.071
Gender (M/F)	146/82	26/27	120/55	0.014
BMI (kg/m^2^), median (IQR)	29.1 (25.9–32.9)	30.8 (27.4–33.4)	29.0 (25.4–32.3)	0.032
Waist (cm), median (IQR)	99 (98–108)	102 (94–110)	98 (90–106)	0.013
T2DM, *n* (%)	76 (33.3%)	25 (47.2%)	51 (29.1%)	0.020
Hypertension, *n* (%)	91 (39.9%)	18 (34.0%)	73 (41.7%)	0.340
ALT (U/L), median (IQR)	45 (30–70)	56 (35–86)	41 (29–68)	0.060
AST (U/L), median (IQR)	30 (22–42)	39 (28–53)	27 (21–39)	0.002
γGT (U/L), median (IQR)	45 (26–92)	62 (38–107)	41 (24–84)	0.012
Platelets (×10^9^/L), median (IQR)	227 (190–270)	226 (197–268)	227 (189–270)	0.953
Albumin (g/dL), median (IQR)	4.4 (4.1–4.6)	4.3 (4.0–4.6)	4.4 (4.2–4.6)	0.652
Total bilirubin (mg/dL), median (IQR)	0.7 (0.5–0.9)	0.7 (0.5–1.0)	0.7 (0.5–0.9)	0.908
Fasting glucose (mg/dL), median (IQR)	94 (86–110)	98 (90–124)	93 (85–106)	0.019
Fasting insulin (mU/L), median (IQR)	13.9 (9.4–23.9)	18.2 (12.6–27.8)	12.6 (8.2–21.4)	0.002
HOMA‐IR, median (IQR)	3.39 (2.04–6.14)	4.46 (2.94–7.94)	2.96 (1.86–5.52)	0.001
Total cholesterol (mg/dL), median (IQR)	184 (161–211)	181 (165–211)	185 (160–211)	0.683
HDL cholesterol (mg/dL), median (IQR)	46 (39–55)	45 (39–53)	46 (39–55)	0.625
Liver stiffness (kPa), median (IQR)	7.5 (6.0–10.3)	10.3 (7.5–15.0)	6.9 (5.7–8.7)	< 0.001
Liver histology				
Steatohepatitis, *n* (%)	165 (72.4%)	53 (100%)	112 (64.0%)	< 0.001
Liver fibrosis, *n* (%)				
F0	65 (28.5%)	0	65 (37.1%)	< 0.001
F1	69 (30.3%)	0	69 (39.4%)	
F2	29 (12.7%)	12 (22.6%)	17 (9.7%)	
F3	33 (14.5%)	25 (47.2%)	8 (4.6%)	
F4	32 (14.0%)	16 (30.2%)	16 (9.1%)	

*Note:* We defined ‘at risk MASH’ as patients with MASH who had a NAFLD Activity Score (NAS) ≥ 4 and a fibrosis stage ≥ 2.

Abbreviations: γGT, gamma‐glutamyl transferase; ALT, alanine aminotransferase; AST, aspartate aminotransferase; BMI, body mass index; HDL, high‐density lipoprotein; HOMA‐IR, homeostasis model assessment of insulin resistance; IQR, interquartile range; MASH, metabolic‐dysfunction associated steatohepatitis; *n*, number; T2DM, type 2 diabetes mellitus.

### 3.2 Serum EVs miRNA Expression in Relation to Steatohepatitis and Liver Fibrosis

**FIGURE 1:**
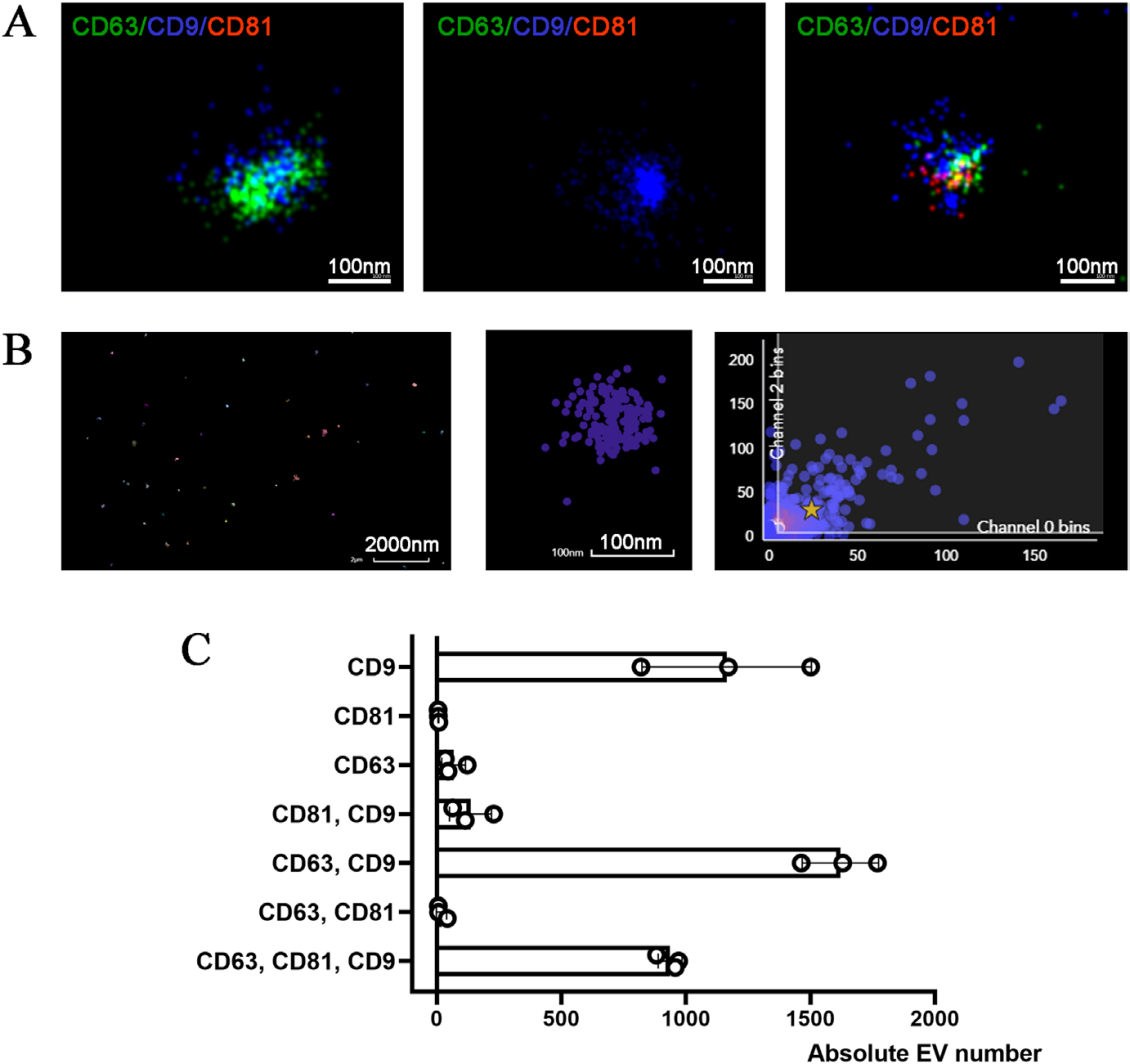
Representative super‐resolution microscopy images of EVs showing double (CD63 and CD9), single (CD9), and triple (CD63, CD9 and CD81) expression of CD63 (green), CD81 (red), and CD9 (blue). The scale bares are below each EV image (100 nm) (A). Representative clustering strategy of EV analysis showing a large field of view (left panel), a selected cluster, and a graph of CD63/CD9 cluster distribution (right panel) (B). Clustering analysis of super‐resolution microscopy images showing the single, double, and triple positive EV fractions expressing the tetraspanins. The analyses were performed in 3 EV preparations using CODI software; the graph shows the mean ± SD of a cumulative analysis of 3 fields for each preparation (C).

The presence of EVs after Exoquick precipitation was confirmed by Super‐resolution microscopy and by NanoSight analyses (Figure 1, Figure S1). Super‐resolution microscopy was performed on 3 representative samples confirming EV size in the range of 100–200 nm and the expression of the classical exosomal markers, tetraspanins (CD63, CD9, and CD81). Results showed that EVs were single, double, or triple positive for tetraspanin expression, with the majority of them being double positive for CD63 and CD9, and single positive for CD9 (Figure 1).

**FIGURE 2:**
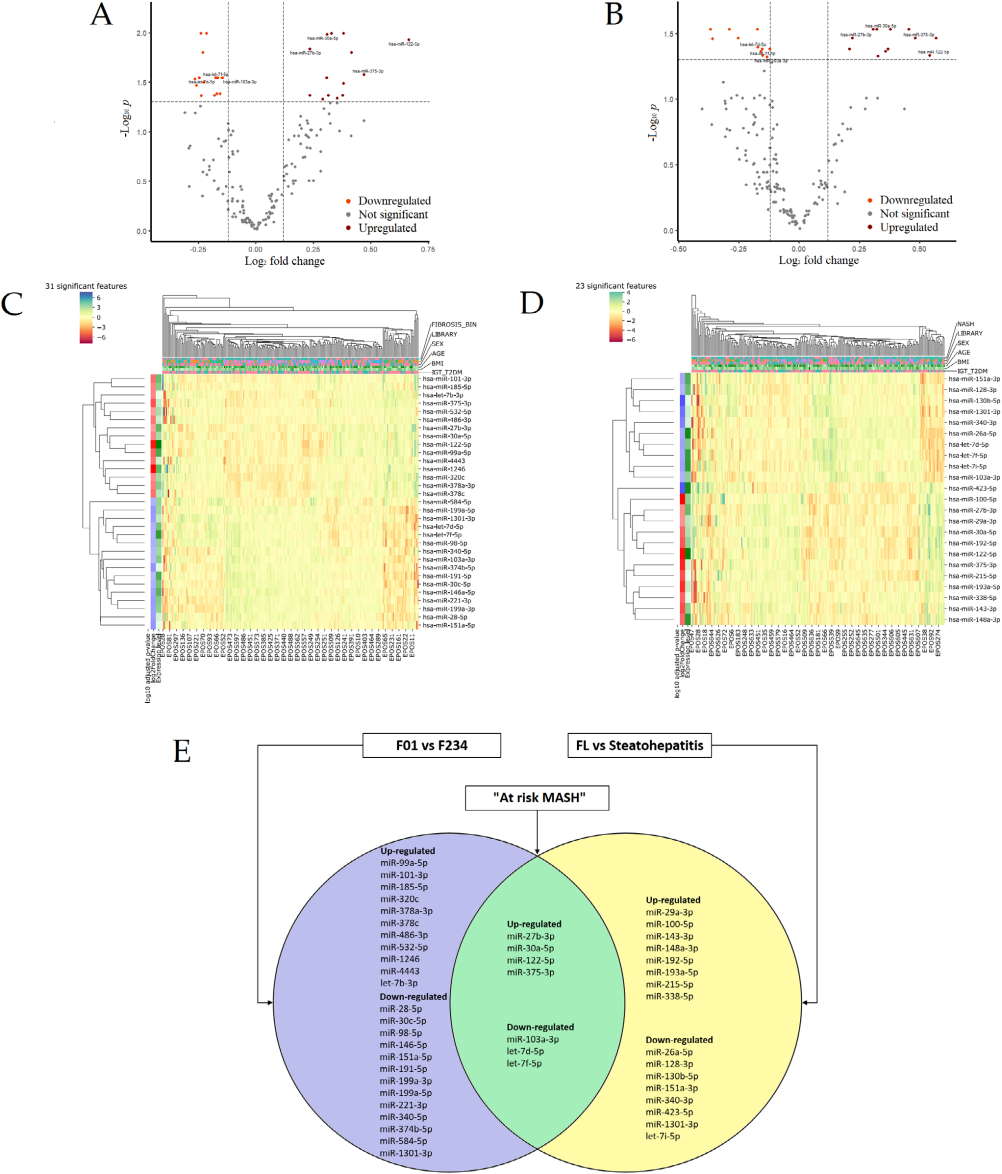
Volcano plots depicting differentially expressed miRNAs according to the presence of significant fibrosis (*F* ≥ 2) (A) and to the diagnosis of steatohepatitis (B). Heatmaps of significantly deregulated miRNAs according to the presence of significant fibrosis (*F* ≥ 2) (C) and to the diagnosis of steatohepatitis (D). Venn diagram depicting deregulated miRNAs according to liver fibrosis and steatohepatitis diagnosis and their intersection in patients ‘at risk MASH’ (E). We defined “at risk MASH” as patients with MASH who had a NAFLD Activity Score (NAS) ≥ 4 and a fibrosis stage ≥ 2. Abbreviations: FL, fatty liver.

We performed a differential expression analysis to find miRNAs associated with the presence of significant fibrosis (*F* ≥ 2) and the diagnosis of steatohepatitis. Following adjustment for library, age, gender, BMI, and T2DM, we observed 23 and 31 differentially expressed miRNAs according to the presence of significant fibrosis (Figure 2A) and the diagnosis of steatohepatitis (Figure 2B), respectively. Hierarchical clustering and heatmaps showed that individuals with *F* ≥ 2 (Figure 2C) and with steatohepatitis (Figure 2D) were almost grouped together. The significantly differentially expressed miRNAs were divided into three groups according to their expression profiles (Figure 2E).

Considering a log_2_ fold change (FC) ≥ 0.5, in subjects with a diagnosis of steatohepatitis, miR‐122‐5p (log_2_ FC = 0.54, adjusted *p* = 0.046) and miR‐100‐5p (log_2_ FC = 0.57, adjusted *p* = 0.034) were the most significantly up‐regulated miRNAs (Table S1). In patients with significant fibrosis (*F* ≥ 2), two miRNAs were up‐regulated: miR‐1246 (log_2_ FC = 0.67, adjusted *p* = 0.012) and once again miR‐122‐5p (log_2_ FC = 0.67, adjusted *p* = 0.012) (Table S2).

Overall, we identified seven deregulated miRNAs in ‘at‐risk MASH’ patients (Figure 2E): miR‐27b‐3p, miR‐30a‐5p, miR‐122‐5p, and miR‐375‐3p resulted consistently up‐regulated, while miR‐103a‐3p, let‐7d‐5p, and let‐7f‐5p were significantly down‐regulated. Next, we investigated the variation of the seven miRNAs according to each histologic feature of steatohepatitis (i.e., liver steatosis, hepatocyte ballooning, and lobular inflammation). Most miRNAs were deregulated according to the degree of hepatocyte ballooning and the presence of lobular inflammation, but only miR‐122‐5p was significantly up‐regulated in parallel to the degree of steatosis (Figure S2).

### 3.3 Relationship Between Serum EVs miRNAs Expression and Metabolic Substrates/Fluxes

We investigated the relationship between the seven above‐selected miRNAs (miR‐27b‐3p, miR‐30a‐5p, miR‐122‐5p, miR‐375‐3p, miR‐103a‐3p, let‐7d‐5p, and let‐7f‐5p) and metabolic flexibility in relation to glucose and lipid fluxes in a subgroup of non‐diabetic MASLD patients who underwent tracer studies (*n* = 54), to assess their potential pathophysiological role in MASLD metabolism, independent of T2DM. The characteristics of this subgroup of patients, along with their metabolic data, are reported in Table S3.

Firstly, we investigated the miRNA's relationship with glucose metabolism. Among the selected miRNAs, miR‐27b‐3p was directly correlated to hepatic insulin resistance (Hep‐IR) (*r* = 0.279, 95% CI: 0.009–0.510, *p* = 0.043), while miR‐103a‐3p showed an inverse correlation with Hep‐IR (*r* = −0.273, 95% CI: −0.506 to −0.003, *p* = 0.048); no miRNA was correlated with glucose clearance (data not shown).

Next, we investigated the correlation between the seven miRNAs and lipid metabolism. We observed an inverse correlation between let‐7d‐5p and let‐7f‐5p with lipolysis by tracers and with FFAs levels. Both let‐7d‐5p and let‐7f‐5p levels progressively decreased according to the amount of IR in the adipose tissue (AT‐IR). No other miRNAs correlated with lipolysis; however, miR‐122‐5p and miR‐375‐3p levels directly correlated with FFAs and significantly increased according to the worsening of AT‐IR (Table S4, Figure S3).

To further characterise lipid profiles in relation to the above‐reported miRNAs, we assessed the composition of FFAs in two‐thirds (39/54) of the tracers study subjects, revealing a significant association with the subclasses of saturated fatty acids (SFAs) and monounsaturated fatty acids (MUFAs). In particular, miR‐122‐5p resulted directly correlated to SFAs (*r* = 0.424, 95% CI: 0.126–0.652, *p* = 0.007) and MUFAs (*r* = 0.384, 95% CI: 0.078–0.624, *p* = 0.016), while miR‐375‐3p was correlated to MUFAs (*r* = 0.436, 95% CI: 0.140–0.661, *p* = 0.006). Conversely, we found an inverse relationship of let‐7d‐5p with SFA (*r* = −0.396, 95% CI: −0.633 to −0.092, *p* = 0.013) and MUFAs (*r* = −0.360, 95% CI: −0.606 to −0.050, *p* = 0.025), as well as of let‐7f‐5p with SFA (*r* = −0.454, 95% CI: −0.673 to −0.162, *p* = 0.004) and MUFAs (*r* = −0.478, 95% CI: −0.690 to −0.192, *p* = 0.002).

Regarding miR‐103a‐3p, miR‐27b‐3p, and miR‐30a‐5p, we did not observe any association with lipolysis, FFA levels, and AT‐IR, suggesting their marginal metabolic effect on lipid metabolism in the context of non‐diabetic MASLD patients.

### 3.4 Gene Expression Studies in Human Samples

**FIGURE 3:**
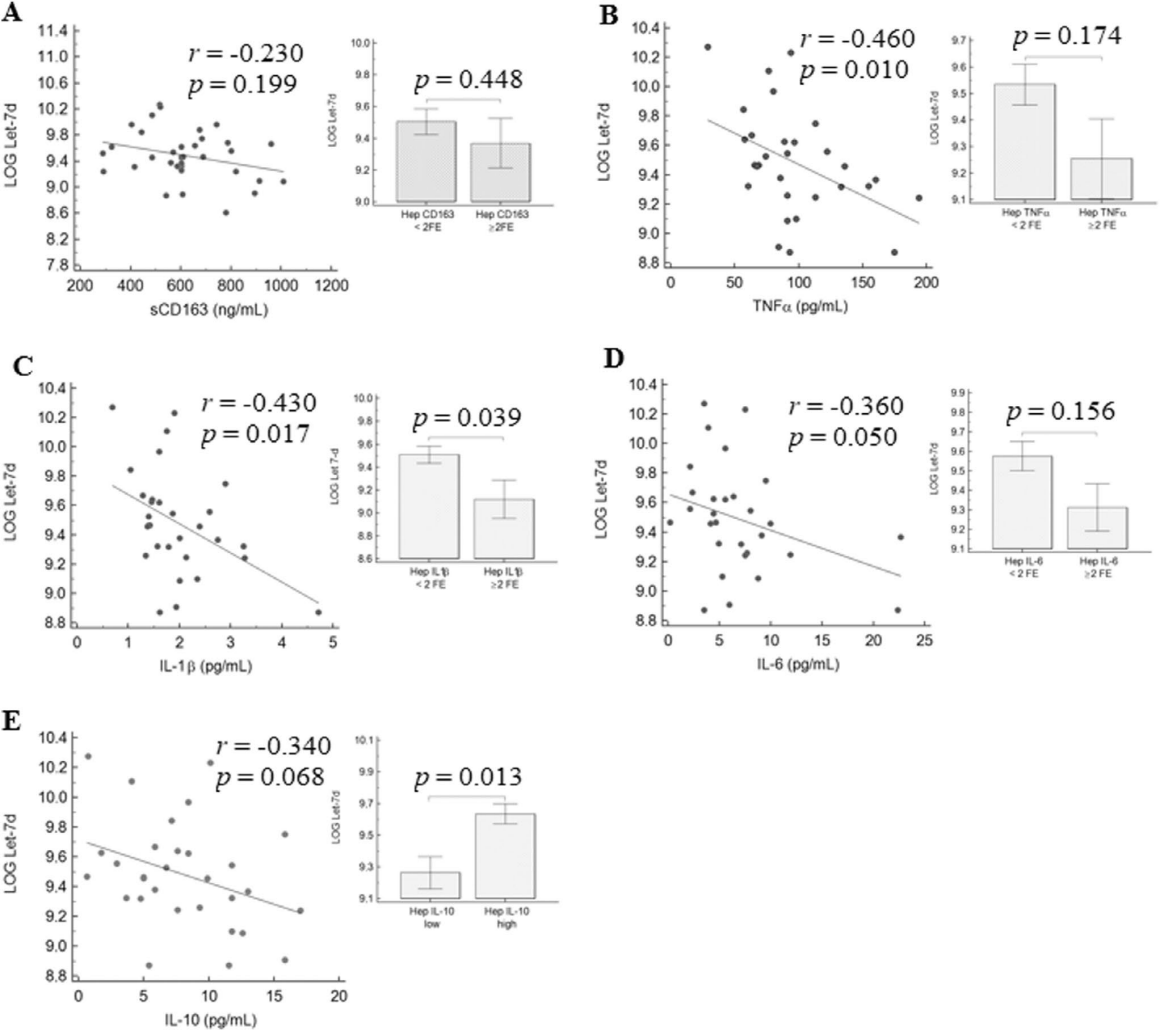
Correlation between let‐7d‐5p and circulating and hepatic expression of CD163 (A), TNFα (B), IL‐1β (C), IL‐6 (D), and IL‐10 (E). *p* values were calculated by Spearman's correlation test or by Mann–Whitney test.

**FIGURE 4:**
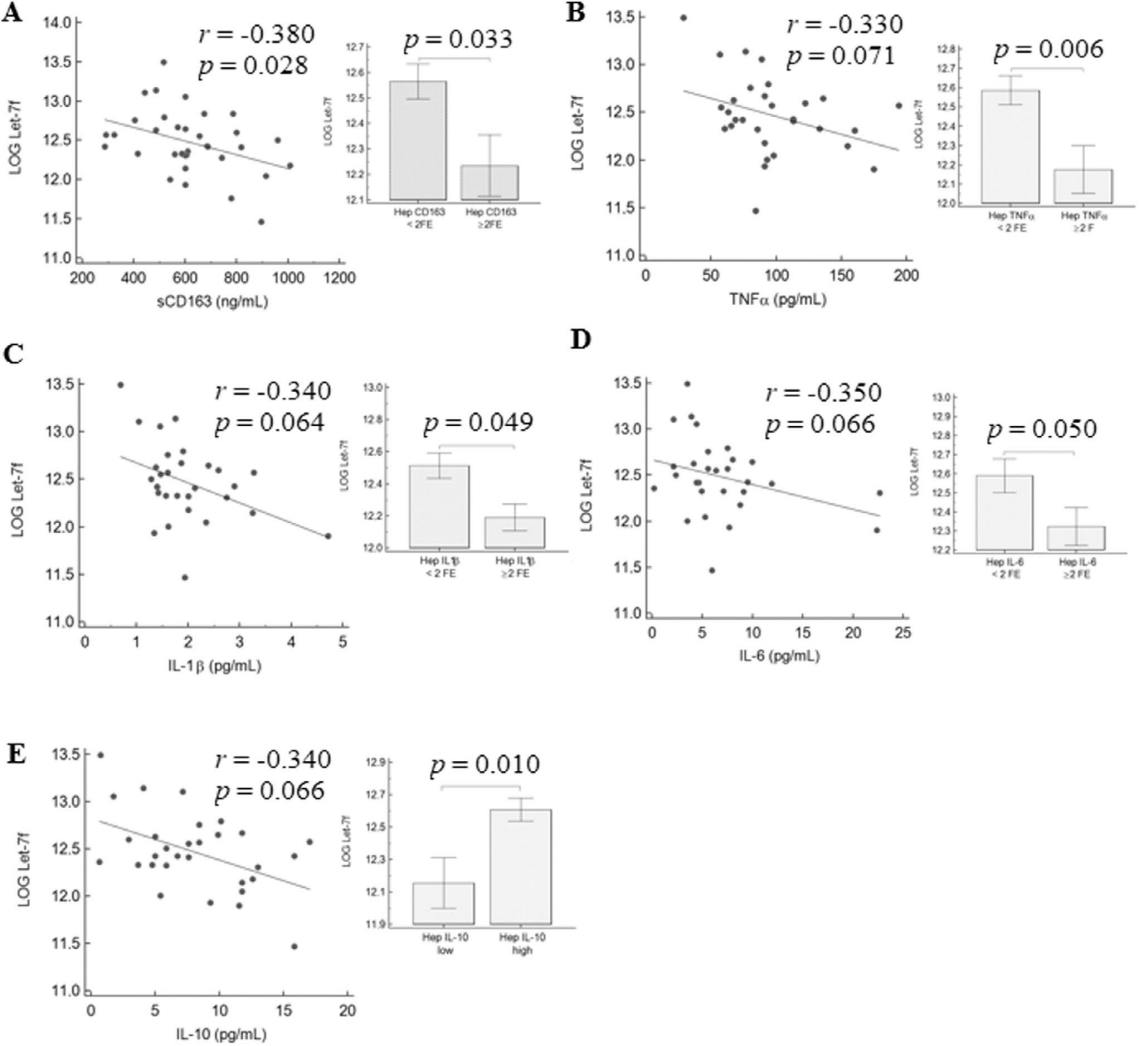
Correlation between let‐7f‐5p and circulating and hepatic expression of CD163 (A), TNFα (B), IL‐1β (C), IL‐6 (D), and IL‐10 (E). *p* values were calculated by Spearman's correlation test or by Mann–Whitney test.

In a subgroup of 30 MASLD patients with available liver tissue, we performed a gene expression study to explore the associations between circulating miRNAs and the hepatic expression of a panel of target genes involved in pro/anti‐inflammatory pathways. Overall, we observed an inverse correlation between circulating let‐7d‐5p and let‐7f‐5p with the hepatic expression of CD163, tumour necrosis factor (TNF)α, interleukin (IL)‐1β, and IL‐6, and a direct association with IL‐10. The same trend was observed between serum let‐7d‐5p and let‐7f‐5p and circulating levels of the corresponding cytokines (Figures 3 and 4). No correlation was found between miR‐122‐5p and miR‐375‐3p with the hepatic expression of pro/anti‐inflammatory factors. Interestingly, circulating levels of sCD163, IL‐1β, IL‐6, and TNFα were significantly correlated to AT‐IR (Table S5).

Results from target gene and enrichment analysis are reported in the Supporting Information: Results section.

### 3.5 MiRNA Expression in the Animal Model

Based on gene expression results from human samples, we investigated the hepatic expression of miR‐122‐5p, miR‐375‐3p, let‐7d‐5p, and let‐7f‐5p in a mouse model that mimics the conditions of MASLD/MASH. Notably, we observed a significant up‐regulation of miR‐375‐3p (*p* = 0.019), let‐7d‐5p (*p* = 0.030), and let‐7f‐5p (*p* = 0.030) in CDAA‐fed mice compared to CSAA‐fed controls, while miR‐122‐5p (*p* = 0.093) exhibited a trend towards up‐regulation (Figure S4).

## 4 Discussion

In the present study, we investigated the miRNome profile of circulating EVs obtained from patients with biopsy‐proven MASLD using high‐throughput sequencing in relation to: 1. histologic liver damage; 2. in vivo metabolic fluxes (glucose and lipid) evaluated by tracer studies and 3. circulating and hepatic pro‐inflammatory cytokines. We identified a panel of seven miRNAs (miR‐27b‐3p, miR‐30a‐5p, miR‐375‐3p, miR‐122‐5p, miR‐103a‐3p, let‐7d‐5p, and let‐7f‐5p) significantly deregulated in ‘at risk MASH’ patients, currently considered an important target of pharmacotherapy for the increased likelihood of cirrhosis development and risk of liver‐related events [15]. Specifically, these distinct miRNAs were involved in lipid metabolism; the up‐regulated miR‐122‐5p as well as miR‐375‐3p directly correlated to FFAs circulating levels and adipose tissue insulin resistance (AT‐IR), while the down‐regulated let‐7d‐5p and let‐7f‐5p had an opposite trend, showing an inverse correlation with FFAs and AT‐IR. In addition, let‐7d‐5p and let‐7f‐5p were inversely related to both the circulating and hepatic expression of pro‐inflammatory cytokines, which had a positive association with AT‐IR.

MiR‐122‐5p is a liver‐specific miRNA accounting for more than 70% of the entire hepatic miRNA pool [16]. Pre‐clinical studies showed that FFAs increase hepatic secretion of miR‐122 that promotes a switch from fatty acid oxidation to triglycerides synthesis both in the liver and in the adipose tissue [17]. In agreement with these and previous findings [18], we observed that miR‐122‐5p levels in serum EVs significantly increased according to the worsening of AT‐IR and were up‐regulated in patients with steatohepatitis, in those with significant fibrosis, and consequently in “at risk MASH” subjects.

Along with miR‐122‐5p, we also identified up‐regulated miR‐375‐3p in significant liver fibrosis and steatohepatitis. In our series, miR‐375‐3p was significantly correlated with circulating FFAs, AT‐IR, steatohepatitis, and liver fibrosis. In addition, in our mouse model mimicking the conditions of MASLD/MASH, we observed an intrahepatic up‐regulation of miR‐375‐3p in CDAA‐fed mice compared to CSAA‐fed controls. These results strongly suggest that also miR‐375‐3p can be mechanistically involved in the metabolic derangements that lead to MASLD. Previous studies showed that miR‐375 was highly expressed in pancreatic islets of humans and mice, where it was involved in the regulation of glucose‐mediated insulin secretion by β cells [19]. Moreover, it has been shown that miR‐375 inhibition leads to increased expression of adiponectin, reduction of lipid accumulation, and pro‐inflammatory cytokines expression in palmitic acid‐induced HepG2 cells [20]. We can hypothesise that the up‐regulation of miR‐122‐5p and miR‐375‐3p following increased flux of FFAs may promote de novo lipogenesis (DNL) through two distinct pathways: miR‐122‐5p via inhibiting the liver kinase B1 (LKB1)/AMP‐activated protein kinase (AMPK) pathway by targeting Sirt1 [21], while miR‐375‐3p via inhibiting adiponectin receptor 2 (AdipoR2)/peroxisome proliferator‐activated receptor (PPAR)‐α pathway [20, 22]. As a result, increased DNL induces liver steatosis, promotes liver damage, and furthers disease progression.

The let‐7 miRNA family plays a key role in the modulation of inflammatory response regulating the expression of different pro‐inflammatory cytokines and chemokines such as granulocyte‐macrophage colony‐stimulating factor (GMC‐SF), interferon (IFN)γ, IL‐1β, IL‐6, IL‐8, monocyte chemoattractant protein (MCP)‐1 and TNFα [23, 24]. Previous studies showed that the overflow of FFAs is responsible for the activation in the liver of nuclear factor kappa B (NF‐kB), a transcription factor involved in the regulation of several biological processes, including immune response and inflammation [25]. NF‐κB, in turn, triggers the activation of Lin28B, an inhibitor of microRNA processing, leading to the downregulation of let‐7 miRNAs in vitro [26]. Consistent with these findings, our study revealed an inverse correlation between circulating let‐7d‐5p and let‐7f‐5p with lipolysis, FFAs (mainly SFA and MUFA), and AT‐IR. Conversely, we observed an upregulation of let‐7d‐5p and let‐7f‐5p expression within the liver in the MASLD/MASH mouse model. This observation aligns with the results of Infante‐Menéndez et al. [27], who described a dual expression pattern for let‐7d‐5p, characterised by overexpression in the liver and downregulation in circulation. This duality may be attributed to either reduced secretion or increased sequestration of let‐7d‐5p within cellular compartments, limiting its release into the bloodstream.

In addition, let‐7 is known to directly inhibit the expression of IL‐6 by binding to the 3' untranslated region (UTR) of the target mRNA. Activation of NF‐κB and the subsequent downregulation of circulating let‐7 result in elevated IL‐6 levels [26]. In our study, both serum levels and hepatic expression of IL‐6, along with IL‐1β, TNF‐α, and sCD163 (a specific marker of macrophage activation) [28], showed an inverse correlation with circulating let‐7d‐5p and let‐7f‐5p in human samples. Moreover, these pro‐inflammatory cytokines were positively associated with adipose tissue insulin resistance (AT‐IR).

**FIGURE 5:**
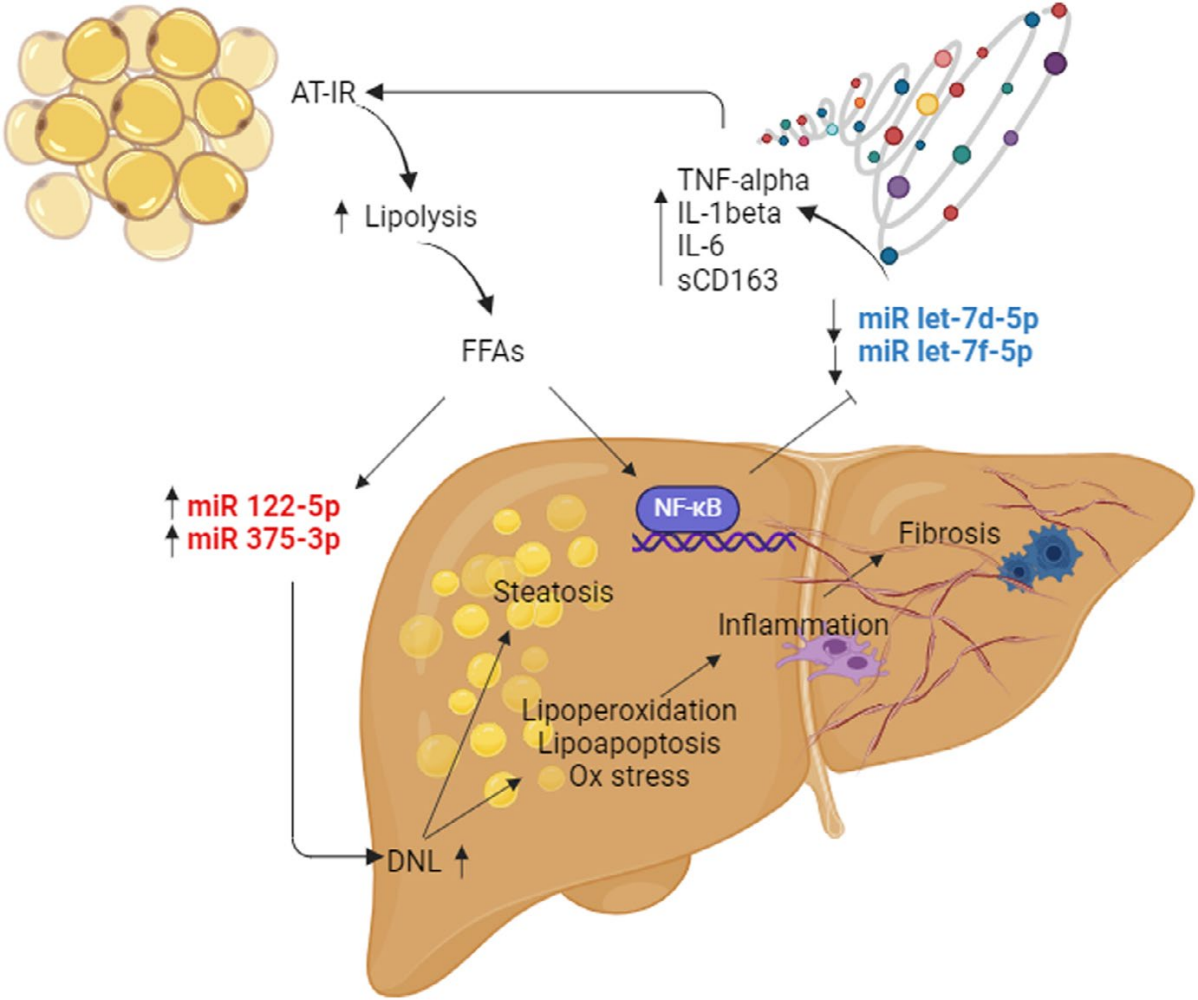
Our suggested model for liver disease progression in patients with MASLD involving miR‐122‐5p, miR‐375‐3p, let‐7d‐5p, and let‐7f‐5p. The increased flux of FFAs due to AT‐IR induces the expression of miR‐122‐5p and miR‐375‐3p that in turn promote de novo lipogenesis (DNL), inducing liver steatosis, inflammation, and fibrosis. On the other side, FFAs induce a NF‐kB‐mediated down‐regulation of let‐7d‐5p and let‐7f‐5p, leading to an increase of pro‐inflammatory cytokines that further enhance AT‐IR. Abbreviations: AT‐IR, adipose tissue insulin resistance; DNL, de novo lipogenesis; FFAs, free fatty acids; IL, interleukin; NF‐kB, nuclear factor kappa B; TNFα, tumour necrosis factor alpha. Created with BioRender.com.

Taken together, our and previous findings suggest that low levels of let‐7 reflect a FFAs‐driven downregulation of the two miRNAs, coupled with upregulation of pro‐inflammatory pathways involved in the progression of liver damage in MASLD. Specifically, the increased flux of FFAs due to AT‐IR induces the expression of miR‐122‐5p and miR‐375‐3p, which, in turn, promote de novo lipogenesis (DNL), leading to liver steatosis, inflammation, and fibrosis. On the other hand, FFAs also induce NF‐κB‐mediated downregulation of circulating let‐7d‐5p and let‐7f‐5p, further promoting the expression of pro‐inflammatory cytokines, which intensify AT‐IR and perpetuate the cycle of inflammation and liver damage (Figure 5). It seems reasonable to assume that members of the let‐7 family, along with miR‐375‐3p and miR‐122‐5p, serve as key modulators of inflammation and lipid metabolism in MASLD, highlighting their potential as therapeutic targets. As a matter of fact, preclinical studies have already demonstrated promising results with the use of miRNA mimics and inhibitors to target metabolic pathways associated with liver steatosis, insulin resistance, and fibrosis [29].

Finally, our results partially align with those reported by Johnson and colleagues [30], who observed significantly reduced circulating levels of let‐7d‐5p and let‐7f‐5p, along with increased expression of miR‐122‐5p in patients with MASLD compared to healthy subjects, thereby further supporting our findings. Nevertheless, it should be noted that in our study we investigated the expression of miRNAs packaged in EVs, mainly exosomes, thus restricting the analysis to those miRNAs actively released into the circulation. The exclusion of miRNAs passively released into the bloodstream following cell death allowed us to gain an unbiased insight into the pathophysiology of liver damage and to unveil novel miRNAs with diagnostic/prognostic potential.

In conclusion, in a cohort of well‐characterised patients with histological MASLD, we obtained a comprehensive profile of miRNAs contained within EVs, providing further evidence of the involvement of several miRNAs in the pathophysiological mechanisms underlying liver damage. In particular, our results suggest an intertwined connection of miR‐122‐5p, miR‐375‐3p, and let‐7 family with lipid derangements and inflammatory pathways. Further studies are warranted to better define the pathophysiological role of these miRNAs in the progression of steatohepatitis as well as the onset of liver‐related complications and to find new potential therapeutic strategies.

## Author Contributions

**Gian Paolo Caviglia:** investigation, writing – original draft, software, formal analysis, data curation, methodology, validation, visualization. **Elisabetta Casalone:** investigation, writing – original draft, methodology, validation, visualization, software, formal analysis, data curation. **Chiara Rosso:** investigation, methodology, software, formal analysis, data curation, writing – review and editing. **Serena Aneli:** methodology, software, formal analysis, investigation. **Alessandra Allione:** methodology, data curation, formal analysis, writing – review and editing. **Fabrizia Carli:** methodology, formal analysis, data curation, investigation. **Cristina Grange:** investigation, formal analysis. **Angelo Armandi:** data curation, software. **Chiara Catalano:** data curation, investigation. **Giovanni Birolo:** formal analysis, software. **Beatrice Foglia:** resources. **Davide Giuseppe Ribaldone:** investigation. **Amalia Gastaldelli:** formal analysis, investigation, writing – review and editing. **Giuseppe Matullo:** conceptualization, writing – review and editing, supervision, project administration, funding acquisition. **Elisabetta Bugianesi:** conceptualization, funding acquisition, writing – review and editing, project administration, supervision.

## Ethics Statement

All patients signed a written informed consent for the collection of personal data and for the use of blood samples for research purposes. This study was conducted according to the guidelines of the Declaration of Helsinki and approved by the Institutional Ethics Committee of A.O.U. Città della Salute e della Scienza di Torino (CEI/522, 17 November 2015).

## Conflicts of Interest

The authors declare no conflicts of interest.

## Supporting information


Data S1.

## Data Availability

The data that support the findings of this study are available from the corresponding author upon reasonable request.
