# Supplementary material for: Extracellular Vesicles miRNome Profiling Reveals miRNAs Engagement in Dysfunctional Lipid Metabolism, Chronic Inflammation and Liver Damage in Subjects With Metabolic Dysfunction‐Associated Steatotic Liver Disease
Source: Aliment Pharmacol Ther. 2025 Apr 10;62(1):22–32. doi: 10.1111/apt.70150 (PMC12151544; doi:10.1111/apt.70150)

**Extracellular vesicles miRNome profiling reveals miRNAs engagement in dysfunctional lipid metabolism, chronic inflammation and liver damage in subjects with metabolic dysfunction-associated steatotic liver disease**

Gian Paolo Caviglia, Elisabetta Casalone, Chiara Rosso, Serena Aneli, Alessandra Allione, Fabrizia Carli, Cristina Grange, Angelo Armandi, Chiara Catalano, Giovanni Birolo, Beatrice Foglia, Davide Giuseppe Ribaldone, Amalia Gastaldelli, Giuseppe Matullo, Elisabetta Bugianesi

Table of contents

SUPPLEMENTARY METHODS……………………………………………………………………2

SUPPLEMENTARY METHODS TABLE 1………………………………………………..……….7

SUPPLEMENTARY RESULTS………………………………………………………….………….8

SUPPLEMENTARY RESULTS TABLE 1………………………………………………………….9

SUPPLEMENTARY RESULTS TABLE 2………………………………………………………...14

SUPPLEMENTARY RESULTS FIGURE 1…………………………………………………….…29

SUPPLEMENTARY RESULTS FIGURE 2……………………………………………………….30

SUPPLEMENTARY REFERENCES………………………………………………………………31

TABLE S1……………………………………………………………...…………………………...33

TABLE S2…………………………………………………………………………………...……...35

TABLE S3……………………………………………………………………………………...…...37

TABLE S4……………………………………………………………………………………...…...39

TABLE S5……………………………………………………………………………………...…...40

FIGURE S1…………………………………………….…….……………………………...……...41

FIGURE S2…………………………………………….…….………………………………...…...42

FIGURE S3…………………………………………….…….……………………………...……...43

FIGURE S4…………………………………………….…….……………………………...……...44

**Supplementary Materials and Methods**

*Nanoparticle tracking analysis*

EV concentration was measured by Nanosight NS300 (Malvern Instruments Ltd., Malvern, UK) equipped with a 488 nm laser module that utilizes Brownian motion and refraction index. Samples were diluted 1:200 in physiologic solution filtered with 100 nm pore size. For each sample, 3 videos of 30 s at camera level 15 and threshold 5 were captured. All samples were characterized with NTA 3.2 Analytical software (Malvern Instruments Ltd., Malvern, UK).

*Super-resolution microscopy*

Super-resolution microscopy acquisition of EVs isolated from serum was obtained using Nanoimager S Mark II microscope from ONI (Oxford Nanoimaging, Oxford, UK) equipped with a 100x, 1.4NA oil immersion objective, an XYZ closed-loop piezo 736 stage, and triple emission channels split at 640, 488 and 555 nm. EV profiler Kit (ONI) was used to perform the experiments following the manufacturer’s protocol. The Kit contains fluorescent antibodies anti-CD9-488, CD63-568, and CD81-647 and the imaging buffer. Images were taken in dSTORM mode acquired sequentially in total reflection fluorescence (TIRF) mode. Single-molecule data was filtered using NimOS software (v.1.18.3, ONI). Data has been processed with the Collaborative Discovery (CODI) online analysis platform [www.alto.codi.bio](http://www.alto.codi.bio/) from ONI and the drift correction pipeline version 0.2.3 was used [1].

*Glucose and lipid metabolism studies*

*In vivo* clinical studies for assessing glucose and lipid metabolism were performed in a subgroup of MASLD patients (*n* = 54) by using stable isotopes within 3 months of liver biopsy. In this specific subgroup, patients with T2DM had been excluded to rule out the confounding impact of T2DM. Primed-continuous infusion of [6,6-^2^H_2_]glucose (bolus 22 µmol/kg, infusion rate 0.22 µmol/kg min) and [^2^H_5_]glycerol (bolus 1.5 μmol/kg, infusion rate 0.1 μmol/kg/min) was administered for 2 h in fasting conditions (equilibration period) to assess endogenous glucose production (EGP) and lipolysis by the rate of appearance (Ra) of glycerol, as previously described.^2,3^ Tracer enrichment of [6,6-^2^H_2_]glucose and [^2^H_5_]glycerol were determined by gas chromatography–mass spectrometry (GC-MS) system (Agilent Technology GC7890-MS5975, Santa Clara, CA, USA) after derivatization with acetic anhydride and pyridine 1:1, selectively monitoring ions at mass to charge ratios of 200-205 and 145-148 for glucose and glycerol respectively. Data were expressed as tracer/trace ratios (TTR) as previously described [2,3]. Metabolic fluxes were calculated as infusion rate/TTR. Adipose tissue IR was calculated as the product of FFAs x fasting insulin (AT-IR) while Hepatic IR (Hep-IR) was derived from EGP x fasting insulin [2].

*Free fatty acids determination*

Concentrations of FFAs were determined by enzymatic colorimetric assays (WAKO diagnostic,

Richmond, VA, USA) in plasma samples collected in EDTA and kept in ice until centrifugation and storage. FFAs composition was analyzed by GC-MS (Agilent Technology GC7890-MS5975, Santa Clara, CA, USA) after derivatization by t-Butyldimethylsilyl (tBDMS) (Merck Darmstadt, Germany).

*Gene expression studies in humans*

In a subgroup of 30 patients with available liver biopsy, we performed a gene expression study to assess the hepatic expression of a panel of pro-inflammatory factors. Biopsies were collected in RNAlater (Ambion Inc., Austin, TX) and stored at -80°C until processing. Total RNA extraction was performed using TRIzol reagent (Invitrogen, CA). For each sample, 500 ng of total RNA was treated with deoxyribonuclease I (Invitrogen, CA) before reverse transcription. Then, a retro-transcription reaction was performed with random primers and SuperScript® II Reverse Transcriptase (Invitrogen, CA). Glyceraldehyde 3-phosphate dehydrogenase (GAPDH) was used as housekeeping gene. All the primers for the target genes were designed with PRIMER3 and reported in Supplementary Methods Table 1. A cDNA derived from a patient without features of MASH was used as a calibrator. Quantitative polymerase chain reaction (qPCR) was performed using the SsoAdvancedTM Universal SYBR Green Supermix (Bio-Rad Laboratories, Hercules, CA) according to the following amplification protocol: 98°C for 30 seconds for polymerase activation, 95°C for 15 seconds for cDNA denaturation followed by 39 cycles of 60°C for 30 seconds for annealing and extension. All samples and negative controls (no template cDNA) were run in duplicate and at least two separate measurements were performed for both the housekeeping and target genes. The relative expression of the target genes compared to the calibrator sample was calculated using the following formula: 2-ΔCt. Results were expressed as fold expression (FE) increase over the calibrator sample. The melting curve analysis was performed to assess the specificity of the amplified PCR products. Gene expression analysis was performed by CFX ManagerTM Software (Bio-Rad Laboratories, CA).

*Gene expression studies in mice*

At the time of death, the livers of the animals were collected and stored at -80°C until

processing. RNA extraction was performed using TRIzol reagent (Biorad, CA). Specific miRNA intended retro-transcription reaction was performed with miRCURY LNA RT Kit (Qiagen, Germany), and subsequent qPCR was performed with miRCURY LNA SYBR Green PCR Kit and specific miRCURY LNA miRNA PCR Assay primers (mmU-miR-122-5p, mmU-miR-375-3p, mmU-let-7d-5p, mmU-let-7f-5p, U6 snRNA-v2) (Qiagen, Germany) following manufacturer instructions.

*Cytokines and inflammatory biomarkers*

Circulating sCD163 was measured by the ELISA method (Quantikine® ELISA Human CD163, R&D Systems, Minneapolis, MN, USA); results were reported in ng/mL. A cytokine panel including interleukin (IL)-1β, IL-6, IL-10, monocyte chemoattractant protein (MCP)-1 and tumor necrosis factor (TNF)α, was measured in serum samples by Bio-Plex® Multiplex Immunoassay (Bio-rad Laboratories, Pleasanton, CA, USA) on Luminex® 200 system (Luminex Corporation, Austin, TX, USA). Cytokine results were reported in pg/mL.

*Histological assessment*

All liver biopsies were examined by a local expert liver pathologist blinded to patient’s clinical information. The average length of liver tissue was 25 mm (range 14–45 mm), with a minimum of 11 portal tracts. Histological features of MASLD, such as steatosis, inflammation, ballooning degeneration, and fibrosis, were assessed and scored according to Kleiner et al [4]. MASLD was defined by the presence of steatosis in >5% of hepatocytes; the concomitant presence of steatosis, hepatocyte ballooning, and lobular inflammation was required for a diagnosis of steatohepatitis [5]. For this study we will keep the definition of “at risk MASH” as patients who had a NAFLD Activity Score (NAS) ≥4 and a fibrosis stage ≥2 [6].

*MiRNA targets enrichment analysis*

The validated target genes of selected deregulated miRNAs were retrieved with MultiMiR Bioconductor’s package from Mirecords, Mirtarbase, and Tarbase databases [7]. The analysis was performed separately for up-regulated and down-regulated miRNAs, and we selected the genes that were validated in at least two of the three databases. Gene Ontology-Biological Process enrichment analysis was performed using ShinyGO v0.741, showing the top 50 significant terms (*p* value < 0.05 for False Discovery Rate, FDR) [8]. FDR was controlled by Benjamini-Hochberg procedure, which adjusts the *p* values to account for the number of comparisons made. The Kyoto Encyclopedia of Genes and Genomes (KEGG) enrichment analysis was performed using PathfindR [9], setting a minimum of five genes in each term.

*Data analysis from miRNA sequencing*

Raw reads adapter clipping was performed using the Cutadapt software (version 1.18) [10]; reads longer than 14 nucleotides were mapped to a small non-coding (sncRNA) reference with the bwa alignment software (version 0.7.17-r1188) [11], using the mem algorithm and a seed length of 10. Then, we retained the alignments without mismatches or indels and we selected those with the highest quality to assign each read to a unique sncRNA. Thus, miRNAs were quantified for each sample and then merged into a single count matrix, setting missing miRNAs to zero. MiRNAs whose counts were less than 20 in more than half of the samples were discarded. Differential expression analysis was performed with the DESeq2 Bioconductor’s package (version 1.22.2) [12]. For each model, samples with missing covariates were dropped. MiRNAs were considered significantly associated with a condition or a trend if their *p*-value, after adjustment for multiple testing by FDR, was below the 0.05 threshold.

**Supplementary Methods Table 1 Primer sequences used for the gene expression study.**

|  | **Forward 5’-3’** | **Reverse 5’-3’** |
| --- | --- | --- |
| IL-1β | GGACAAGCTGAGGAAGATGC | TCCATATCCTGTCCCTGGAG |
| IL-6 | TCTCCACAAGCGCCTTCG | CTCAGGGCTGAGATGCCG |
| IL-10 | CCCCAACCACTTCATTCTTG | TCCCAAAGTGCTGGGATTAC |
| TNFα | TCAGGATCATCTTCTCGAACC | GAGTCCTTCTCACATTGTCTC |
| CD163 | CGGCTGCCTCCACCTCTAAGT | ATGAAGATGCTGGCGTGACA |
| GAPDH | GAAGGTGAAGGTCGGAGT | GAAGATGGTGATGGGATTTC |

All the primers were designed using PRIMER3. Abbreviations: cluster of differentiation 163 (CD163); glyceraldehyde-3-phosphate dehydrogenase (GAPDH); interleukin (IL)-1β; interleukin (IL)-6; interleukin (IL)-10; tumor necrosis factor-alpha (TNFα).

**Supplementary Results**

*Target genes and enrichment analyses*

We used the multiMiR Bioconductor’s package to retrieve validated target genes of the identified deregulated miRNAs associated with “at-risk MASH”. We identified 562 and 1011 experimentally validated targets for the down- and up-regulated miRNAs, respectively.

Concerning down-regulated miRNAs (i.e., miR-103a-3p, let-7d-5p, and let-7f-5p), the results of Gene Ontology enrichment analysis of their target genes identified biological processes belonging to the regulation of cellular metabolic and biosynthetic process, cell cycle, cell death, and genes involved in transcriptional regulation (Supplementary Results Figure 1). Analysis of KEGG pathway enrichment revealed several intriguing terms related to Hippo and p53 signaling, as well as to cancers, including hepatocellular carcinoma (HCC) (Supplementary Results Table 1).

On the other hand, the target genes of the up-regulated miRNAs were mainly involved in the regulation of cellular metabolic processes, cell cycle, cell death, apoptotic processes, and protein transport (Supplementary Results Figure 2). The results of the top KEGG pathway analysis were reported in Supplementary Results Table 2; the genes were significantly grouped in pathways related to proteoglycans in cancer, adherens junction, protein processing in endoplasmic reticulum, mitogen-activated protein kinases (MAPK) signaling, mitophagy, spliceosome, non-alcoholic fatty liver disease, lipid, and atherosclerosis.

**Supplementary Results Table 1** Analysis of KEGG pathway enrichment for target genes of down-regulated miRNAs.

| **Term description** | **Fold enrichment** | **Occurrence** | **Lowest**  ***p* value** | **Highest**  ***p* value** | **miRNAs target genes** |
| --- | --- | --- | --- | --- | --- |
| Cell cycle | 4.36 | 10 | 2.20E-14 | 1.40E-07 | CCND1, CDK6, HDAC2, MYC, CDKN1A, CDK1, YWHAZ, YWHAH, WEE1, ESPL1, SMC1A, CDC25A, MCM7 |
| p53 signaling pathway | 6.35 | 10 | 3.30E-09 | 5.30E-08 | MDM4, CDKN1A, CCND1, CDK6, CDK1, TNFRSF10B, PMAIP1, EI24, THBS1, RRM2, PTEN |
| Hippo signaling pathway | 3.30 | 10 | 1.00E-08 | 4.60E-05 | AMOT, SAV1, LATS1, TGFBR1, WNT3A, FZD6, DVL3, YWHAZ, YWHAH, AXIN2, MYC, CCND1 |
| Breast cancer | 3.70 | 10 | 1.20E-07 | 1.30E-06 | NCOA3, SP1, CCND1, MYC, FGF2, IGF1R, PTEN, CDKN1A, WNT3A, FZD6, DVL3, AXIN2, CDK6 |
| Cushing syndrome | 3.24 | 10 | 1.20E-07 | 2.80E-04 | ADCY9, KMT2D, CDK6, CCND1, AHR, CDKN1A, NR4A1, SP1, WNT3A, FZD6, DVL3, AXIN2 |
| Cellular senescence | 2.95 | 10 | 2.10E-07 | 5.40E-07 | TGFBR1, CDK6, CCND1, CDKN1A, PTEN, LIN52, MYC, CDK1, RAD9A, CDC25A, CALM1 |
| MAPK signaling pathway | 2.00 | 10 | 2.80E-07 | 5.00E-04 | GNG12, FGF2, PDGFB, IGF1R, ELK4, MYC, TGFBR1, TAB2, STK4, MAP3K7, MAP2K7, CRKL, DUSP1, NR4A1 |
| Hepatitis B | 1.81 | 10 | 2.80E-07 | 5.70E-06 | TGFBR1, CDKN1A, MYC, YWHAZ, TAB2, MAP3K7, MAP2K7 |
| Non-small cell lung cancer | 3.47 | 10 | 3.90E-07 | 3.10E-05 | CDK6, CCND1, STK4, CDKN1A, KIF5B, EML4 |
| Hepatitis C | 1.89 | 10 | 6.10E-07 | 1.90E-05 | SOCS3, YWHAZ, YWHAH, CDKN1A, CDK6, CCND1, MYC |
| Viral carcinogenesis | 2.02 | 10 | 7.10E-07 | 6.30E-05 | YWHAZ, YWHAH, CDKN1A, CDK1, CCND1, CDK6, HDAC2, PMAIP1 |
| Glioma | 3.88 | 10 | 9.90E-07 | 2.10E-06 | PDGFB, IGF1R, CALM1, PTEN, CDKN1A, CCND1, CDK6 |
| Chronic myeloid leukemia | 3.83 | 10 | 9.90E-07 | 2.10E-06 | CRKL, MYC, CDKN1A, CCND1, CDK6, TGFBR1, HDAC2 |
| Epstein-Barr virus infection | 1.65 | 10 | 9.90E-07 | 2.10E-06 | TAB2, MAP3K7, HDAC2, MYC, CDKN1A, MAP2K7, CCND1, CDK6 |
| Bladder cancer | 5.07 | 10 | 1.20E-06 | 2.90E-06 | MYC, DAPK1, CDKN1A, CCND1, THBS1 |
| Transcriptional misregulation in cancer | 2.66 | 10 | 1.20E-06 | 9.80E-06 | HDAC2, MYC, CCNT1, CCNT2, RUNX2, HMGA2, IGF1R, CDKN1A, ELK4, MYCN, SP1 |
| Hepatocellular carcinoma | 2.97 | 10 | 3.40E-06 | 6.90E-06 | IGF1R, PTEN, MYC, CDKN1A, CDK6, CCND1, TGFBR1, WNT3A, FZD6, DVL3, AXIN2, SMARCC1 |
| Pathogenic Escherichia coli infection | 2.14 | 10 | 5.20E-06 | 4.90E-03 | ARF6, ACTR2, MYO5A, MYH9, WASL, TUBB4A, MAP3K7, TAB2, TNFRSF10B, ABCF2 |
| Melanoma | 4.04 | 10 | 6.30E-06 | 2.40E-05 | FGF2, PDGFB, IGF1R, CCND1, PTEN, CDKN1A, CDK6 |
| Autophagy - animal | 3.89 | 10 | 8.20E-06 | 8.20E-06 | IGF1R, PTEN, BNIP3, ATG9A, SMCR8, RRAGC, CAMKK2, MAP3K7, DAPK1, MTMR3, MTMR4, GABARAPL1, LAMP2 |
| Adherens junction | 3.78 | 10 | 1.10E-05 | 2.00E-05 | WASL, VCL, CSNK2A1, IGF1R, TGFBR1, MAP3K7 |
| Human immunodeficiency virus 1 infection | 2.04 | 10 | 1.10E-05 | 2.10E-05 | CRKL, CALM1, GNG5, GNG12, TAB2, MAP3K7, MAP2K7, CUL4A, WEE1, CDK1 |
| FoxO signaling pathway | 3.25 | 10 | 1.10E-05 | 2.10E-05 | TGFBR1, PRKAB2, STK4, IGF1R, PTEN, CCND1, CDKN1A, BNIP3, GABARAPL1, SOD2 |
| Endometrial cancer | 3.58 | 10 | 1.30E-05 | 2.60E-05 | PTEN, AXIN2, MYC, CCND1, CDKN1A |
| Pancreatic cancer | 2.74 | 10 | 1.60E-05 | 2.80E-05 | CDK6, CCND1, CDKN1A, TGFBR1, RAD51 |
| Small cell lung cancer | 2.71 | 10 | 1.80E-05 | 6.30E-05 | CDKN1A, MYC, CDK6, CCND1, ITGA2, PTEN |
| Nucleocytoplasmic transport | 2.80 | 10 | 1.80E-05 | 5.90E-04 | NUP155, NUP50, KPNA1, KPNA5, IPO9, XPO7, THOC5 |
| Prostate cancer | 2.14 | 10 | 1.80E-05 | 7.40E-05 | PDGFB, IGF1R, PTEN, CDKN1A, CCND1 |
| Thyroid hormone signaling pathway | 2.06 | 10 | 2.60E-05 | 2.30E-04 | HDAC2, NCOA3, MED13, CCND1, MYC, ATP2A2 |
| Kaposi sarcoma-associated herpesvirus infection | 2.15 | 10 | 3.30E-05 | 1.20E-04 | CDKN1A, CDK6, GNG5, GNG12, MAP2K7, CALM1, FGF2, PDGFB, CCND1, MYC |
| ErbB signaling pathway | 2.50 | 10 | 3.60E-05 | 4.80E-03 | CRKL, ABL2, MAP2K7, MYC, CDKN1A |
| Colorectal cancer | 2.90 | 10 | 4.70E-05 | 1.20E-04 | AXIN2, MYC, CCND1, TGFBR1, PMAIP1, CDKN1A |
| Focal adhesion | 1.89 | 10 | 4.90E-05 | 1.00E-03 | THBS1, ITGA2, PDGFB, IGF1R, ZYX, VCL, PTEN, CRKL, CCND1 |
| TGF-beta signaling pathway | 2.34 | 10 | 5.70E-05 | 2.00E-04 | THBS1, TGFBR1, ACVR2B, SP1, MYC |
| Proteoglycans in cancer | 2.05 | 10 | 6.00E-05 | 2.70E-03 | CCND1, IGF1R, CDKN1A, MYC, THBS1, ITGA2, FZD6, RDX, FGF2, WNT3A |
| Salmonella infection | 2.36 | 10 | 6.40E-05 | 2.00E-04 | DYNC1LI2, TUBB4A, ARL8A, ARL8B, KIF5B, ACTR2, ARF6, WASL, MAP3K7, TAB2, KPNA1, MAP2K7, MYC, TNFRSF10B |
| Oocyte meiosis | 3.14 | 10 | 6.80E-05 | 3.80E-04 | IGF1R, ADCY9, CPEB3, YWHAZ, YWHAH, CDK1, ESPL1, SMC1A, CALM1 |
| Osteoclast differentiation | 2.01 | 10 | 6.90E-05 | 1.60E-03 | IFNGR2, TGFBR1, MAP3K7, TAB2, MAP2K7, SOCS3 |
| Regulation of actin cytoskeleton | 1.94 | 10 | 7.80E-05 | 8.70E-03 | FGF2, PDGFB, ITGA2, GNG12, CRKL, MYH9, WASL, ACTR2, RDX, VCL |
| Chemical carcinogenesis - receptor activation | 1.82 | 10 | 8.40E-05 | 2.30E-03 | CDC25A, CCND1, MYC, FGF2, ADCY9, KLF4, AHR, KPNA1, KPNA5 |
| Human T-cell leukemia virus 1 infection | 1.52 | 10 | 9.40E-05 | 4.30E-04 | MYC, ESPL1, CCND1, CDKN1A, PTEN, TGFBR1, ADCY9, ELK4 |
| Signaling pathways regulating pluripotency of stem cells | 4.19 | 10 | 1.10E-04 | 1.10E-04 | KLF4, MYC, ACVR2B, WNT3A, FZD6, DVL3, AXIN2, FGF2, IGF1R, SMARCAD1, RIF1, PCGF3, HAND1, ZFHX3 |
| Endocytosis | 2.24 | 10 | 1.20E-04 | 3.50E-04 | ARF6, AP2A1, TGFBR1, IGF1R, WASL, ACTR2, CAPZA2, VPS4A, RABEP1, RAB10, KIF5B, RAB11FIP4, GBF1 |
| Human cytomegalovirus infection | 2.05 | 10 | 1.50E-04 | 2.90E-04 | SP1, GNG5, GNG12, CALM1, ADCY9, CCND1, MYC, IL6R, CRKL, CDKN1A, CDK6 |
| Fluid shear stress and atherosclerosis | 2.11 | 10 | 1.80E-04 | 2.20E-03 | CALM1, DUSP1, MAP3K7, MAP2K7, PDGFB, EDN1, ACVR2B |
| Measles | 1.51 | 10 | 2.00E-04 | 3.30E-04 | CSNK2A1, MAP3K7, TAB2, CCND1, CDK6 |
| Parathyroid hormone synthesis, secretion and action | 2.38 | 10 | 2.30E-04 | 2.30E-03 | ADCY9, SP1, PDE4D, MEF2D, CDKN1A, RUNX2 |
| Wnt signaling pathway | 2.81 | 10 | 3.60E-04 | 3.60E-04 | WNT3A, ZNRF3, FZD6, DVL3, CSNK2A1, AXIN2, TLE4, MAP3K7, MYC, CCND1, ROR2 |
| Tight junction | 1.81 | 10 | 3.80E-04 | 7.50E-03 | AMOT, CCND1, RDX, MAP2K7, ACTR2, PRKAB2, MYH9 |
| Shigellosis | 1.93 | 10 | 4.00E-04 | 5.50E-03 | CRKL, ACTR2, VCL, WASL, ARF6, BNIP3, MAP3K7, TAB2, RRAGC, GLMN |
| Phagosome | 1.91 | 10 | 4.70E-04 | 5.00E-02 | ATP6V1F, ATP6V1G1, DYNC1LI2, TUBB4A, LAMP2, ITGA2, THBS1 |
| Lipid and atherosclerosis | 1.36 | 10 | 6.30E-04 | 1.90E-03 | SOD2, MAP2K7, TAB2, MAP3K7, MIB1, CALM1, TNFRSF10B |
| AMPK signaling pathway | 2.82 | 10 | 6.60E-04 | 2.00E-03 | MAP3K7, CAB39, CAMKK2, ADIPOR2, PRKAB2, CCND1, RAB10, IGF1R |
| Rap1 signaling pathway | 1.62 | 10 | 7.50E-04 | 1.10E-02 | CALM1, ADCY9, FGF2, PDGFB, IGF1R, CRKL, SIPA1L2, THBS1 |
| Mitophagy | 2.97 | 10 | 8.40E-04 | 1.20E-03 | GABARAPL1, ATG9A, CSNK2A1, BNIP3, SP1 |
| Ras signaling pathway | 1.85 | 10 | 9.40E-04 | 4.80E-03 | FGF2, PDGFB, IGF1R, GNG5, GNG12, CALM1, STK4, SHOC2, ABL2, ARF6 |
| Alcoholism | 1.96 | 10 | 9.60E-04 | 1.40E-02 | HDAC2, GNG5, GNG12, CALM1, CAMKK2 |
| Gastric cancer | 2.51 | 10 | 1.20E-03 | 1.20E-03 | CDKN1A, WNT3A, FZD6, DVL3, AXIN2, MYC, CCND1, TGFBR1, FGF2 |

From left to right, the table reports the description of the enriched term, the fold enrichment value for the enriched term, the number of iterations that the given term was found to enriched over all iterations (occurrence), the lowest adjusted-*p* value of the given term over all iterations, the highest adjusted-*p* value of the given term over all iterations, and the list of miRNAs target genes.

**Supplementary Results Table 2** Analysis of KEGG pathway enrichment for target genes of up-regulated miRNAs.

| **Term description** | **Fold enrichment** | **occurrence** | **Lowest**  ***p* value** | **Highest**  ***p* value** | **miRNAs target genes** |
| --- | --- | --- | --- | --- | --- |
| Ubiquitin mediated proteolysis | 3.47 | 10 | 1.80E-18 | 3.40E-10 | UBA1, SAE1, UBE2D1, UBE2D3, UBE2H, UBE2L3, UBE2K, UBE3C, SMURF2, ITCH, TRIP12, NEDD4L, UBR5, STUB1, PRPF19, CUL1, SKP2, VHL, SOCS1, SOCS3, CDC20, ANAPC5 |
| Cell cycle | 2.96 | 10 | 1.10E-12 | 3.70E-09 | CDK4, RBL1, CCNE2, CUL1, SKP2, CCNA2, DBF4, YWHAZ, YWHAE, WEE1, ANAPC5, CDC20, BUB3, TP53, PCNA, ORC2, ORC6 |
| Protein processing in endoplasmic reticulum | 3.07 | 10 | 5.10E-12 | 4.00E-08 | SEC62, HSPA5, DNAJC3, CALR, MAN1A2, SEC23A, SEC24B, SEC24A, ERP29, SSR3, DERL1, DNAJA1, DNAJB1, BAG2, RAD23B, NFE2L2, MAPK8, BAX, CAPN2, STUB1, UBE2D1, UBE2D3, CUL1 |
| Proteoglycans in cancer | 2.34 | 10 | 6.70E-11 | 6.20E-10 | CD44, VAV3, NRAS, MAPK1, RAC1, PAK1, GAB1, PIK3CD, SLC9A1, PPP1CC, PPP1R12A, IGF1R, EGFR, CASP3, CTNNB1, MET, THBS1, TP53, ITGA2, FRS2, WNT5A, CTSL |
| Viral carcinogenesis | 3.28 | 10 | 8.10E-11 | 3.20E-08 | NRAS, MAPK1, CREB1, YWHAZ, YWHAE, JAK1, CASP3, PIK3CD, TP53, CCNE2, BAX, TBPL1, GTF2H1, GTF2H2C, GTF2E2, GTF2B, CCNA2, SKP2, RBL1, JUN, PKM, RAC1, CDK4, CDC20, PMAIP1 |
| Adherens junction | 3.60 | 10 | 1.20E-10 | 8.80E-07 | RAC1, WASF1, CTNND1, CTNNB1, IGF1R, MET, EGFR, MAPK1, SNAI1, TGFBR1, NLK |
| Epstein-Barr virus infection | 2.15 | 10 | 2.00E-10 | 4.10E-08 | HLA-DQA1, IFNA1, IFNAR2, JAK1, BAX, CASP3, TAPBP, CALR, TP53, PIK3CD, MAPK8, MAP2K3, JUN, CD44, VIM, RAC1, SKP2, CDK4, CCNA2, CCNE2 |
| MAPK signaling pathway | 1.86 | 10 | 2.00E-10 | 6.50E-09 | GNG12, PPP3CA, PPP3CB, PPP3R1, RASGRP3, NF1, BDNF, EGFR, IGF1R, MET, NRAS, MAPK1, MKNK2, STMN1, PLA2G4A, TGFBR1, RAC1, CASP3, PAK1, TAOK1, MAP2K3, MAPK8, JUN, TP53, NLK |
| Spliceosome | 1.84 | 10 | 4.00E-10 | 4.40E-06 | DHX8, PRPF40A, SF3B3, SNRNP27, PRPF8, PRPF19, HNRNPA1, HNRNPA1L2, HNRNPM, HNRNPU, SRSF1, SRSF7 |
| Mitophagy | 3.71 | 10 | 5.00E-10 | 6.50E-10 | MAPK8, JUN, PINK1, GABARAPL2, MAP1LC3B, BECN1, BCL2L1, BNIP3, NRAS, TP53, BNIP3L, SP1 |
| Hepatitis B | 2.28 | 10 | 5.10E-10 | 1.70E-06 | TGFBR1, MAPK8, YWHAZ, PIK3CD, BAX, CASP3, TP53, CCNA2, CCNE2, JAK1, NRAS, JUN, CREB1, PCNA, MAPK1, MAP2K3, IFNA1 |
| Pancreatic cancer | 3.41 | 10 | 6.40E-10 | 2.30E-08 | PIK3CD, RAC1, BCL2L1, MAPK1, MAPK8, EGFR, JAK1, CDK4, TP53, BAX, TGFBR1, BRCA2 |
| Shigellosis | 2.40 | 10 | 6.90E-10 | 6.50E-09 | CD44, RAC1, WASF1, ARPC5, EGFR, PIK3CD, ARF6, CAST, CAPN2, TP53, BCL2L1, HK1, BAX, BNIP3, PPID, MAPK8, JUN, UBE2D1, UBE2D3, CUL1, MAPK1, CBX3, BECN1, MAP1LC3B |
| p53 signaling pathway | 3.90 | 10 | 3.30E-09 | 1.30E-04 | MDM4, TP53, CDK4, CCNE2, TNFRSF10B, BAX, PMAIP1, BCL2L1, CASP3, THBS1, SERPINB5, RRM2, CCNG1 |
| Hepatocellular carcinoma | 2.57 | 10 | 4.70E-09 | 1.20E-08 | EGFR, IGF1R, SHC1, NRAS, MAPK1, PIK3CD, BCL2L1, TP53, BAX, CDK4, TGFBR1, WNT5A, CTNNB1, NFE2L2, HMOX1, GSTM3, MGST2, MET, GAB1, PBRM1 |
| Kaposi sarcoma-associated herpesvirus infection | 2.80 | 10 | 5.40E-09 | 5.40E-09 | IFNA1, IFNAR2, JAK1, ZFP36, BAX, CASP3, TP53, CDK4, BECN1, MAP1LC3B, GNG2, GNG5, GNG12, NRAS, MAPK1, JUN, PIK3CD, RAC1, MAPK8, CALM3, PPP3CA, PPP3CB, PPP3R1, CTNNB1, CREB1 |
| Colorectal cancer | 3.52 | 10 | 5.80E-09 | 4.20E-08 | CTNNB1, PIK3CD, MAPK1, JUN, RAC1, MAPK8, CASP3, TGFBR1, MLH1, BAX, PMAIP1, TP53, EGFR, NRAS |
| Lipid and atherosclerosis | 2.63 | 10 | 6.50E-09 | 6.50E-09 | LDLR, OLR1, PIK3CD, BAX, BCL2L1, MAPK8, SOD2, RAC1, JUN, MAP2K3, NRAS, MAPK1, MIB1, IFNA1, PPARG, VAV3, HSPA5, NFE2L2, CALM3, PPP3CA, PPP3CB, PPP3R1, TNFRSF10B, CASP3, CASP7, TP53 |
| Human cytomegalovirus infection | 2.42 | 10 | 7.40E-09 | 7.40E-09 | PIK3CD, EGFR, NRAS, MAPK1, SP1, RAC1, IFNA1, GNG2, GNG5, GNG12, CALM3, PPP3CA, PPP3CB, PPP3R1, GNAI2, GNA13, CTNNB1, JAK1, CREB1, TAPBP, CALR, TP53, CDK4, BAX, CASP3 |
| Human T-cell leukemia virus 1 infection | 2.57 | 10 | 1.20E-08 | 8.10E-07 | TRRAP, NRAS, MAPK1, XPO1, CALR, PPP3CA, PPP3CB, PPP3R1, JAK1, BUB3, ANAPC5, CDC20, CDK4, CCNA2, CCNE2, TP53, PIK3CD, MAPK8, JUN, ZFP36, BCL2L1, TGFBR1, HLA-DQA1, TBPL1, CREB1, BAX |
| AGE-RAGE signaling pathway in diabetic complications | 2.18 | 10 | 1.30E-08 | 2.60E-05 | TGFBR1, MAPK1, JUN, MAPK8, RAC1, NRAS, PIK3CD, BAX, CASP3, CDK4 |
| Central carbon metabolism in cancer | 3.76 | 10 | 1.40E-08 | 1.30E-06 | PKM, TP53, SLC1A5, MET, RET, EGFR, NRAS, MAPK1, HK1, IDH1, PIK3CD, SLC7A5 |
| Choline metabolism in cancer | 2.50 | 10 | 1.60E-08 | 1.60E-06 | EGFR, NRAS, MAPK1, MAPK8, PLA2G4A, PIK3CD, RAC1, WASF1, SP1, SLC44A1, JUN |
| Rap1 signaling pathway | 1.69 | 10 | 2.10E-08 | 5.80E-06 | CALM3, ADORA2B, RASGRP3, EGFR, IGF1R, MET, CTNNB1, GNAI2, THBS1, RAC1, VAV3, CTNND1, MAPK1, MAP2K3, PIK3CD, NRAS |
| TNF signaling pathway | 1.95 | 10 | 2.40E-08 | 6.10E-04 | MAPK8, JUN, ITCH, MAP2K3, MAPK1, CREB1, CASP7, CASP3, SOCS3, PIK3CD |
| Breast cancer | 2.37 | 10 | 2.80E-08 | 5.80E-08 | NCOA3, JUN, SP1, IGF1R, EGFR, SHC1, NRAS, MAPK1, PIK3CD, NOTCH2, WNT5A, CTNNB1, TP53, BAX, CDK4, BRCA2 |
| TGF-beta signaling pathway | 2.91 | 10 | 2.80E-08 | 4.50E-04 | THBS1, FBN1, NODAL, BMPR2, TGFBR1, SMURF2, ID4, RBL1, SP1, CUL1, MAPK1, PPP2R1B |
| Ras signaling pathway | 2.11 | 10 | 3.20E-08 | 6.90E-08 | BDNF, EGFR, IGF1R, MET, GAB1, SHC1, RASGRP3, GNG2, GNG5, GNG12, CALM3, NRAS, NF1, RAC1, PAK1, PIK3CD, BCL2L1, MAPK1, PLA2G4A, MAPK8, ABL2, ARF6 |
| Gastric cancer | 2.18 | 10 | 3.20E-08 | 1.70E-05 | TP53, BAX, WNT5A, CTNNB1, EGFR, SHC1, NRAS, MAPK1, PIK3CD, MLH1, CCNE2, TGFBR1, JUP, MET, GAB1 |
| Human immunodeficiency virus 1 infection | 2.76 | 10 | 4.00E-08 | 4.20E-08 | CUL1, GNAI2, PIK3CD, JUN, NRAS, MAPK1, CALM3, PPP3CA, PPP3CB, PPP3R1, GNG2, GNG5, GNG12, RAC1, PAK1, BCL2L1, IFNA1, MAPK8, SAMHD1, MAP2K3, BAX, CASP3, TAPBP, CALR, AP1G1, WEE1 |
| Relaxin signaling pathway | 2.38 | 10 | 4.10E-08 | 7.40E-05 | GNAI2, GNG2, GNG5, GNG12, CREB1, PIK3CD, MAPK1, MMP13, TGFBR1, MAPK8, JUN, NRAS, SHC1, EGFR |
| Epithelial cell signaling in Helicobacter pylori infection | 3.09 | 10 | 4.20E-08 | 3.00E-06 | MET, EGFR, ADAM10, RAC1, PAK1, MAPK8, JUN, CASP3, ATP6V1B2, ATP6V1C1 |
| Osteoclast differentiation | 2.61 | 10 | 4.90E-08 | 6.90E-05 | MAPK1, PIK3CD, TGFBR1, MAPK8, JUN, RAC1, PPP3CA, PPP3CB, PPP3R1, CREB1, PPARG, IFNAR2, JAK1, SOCS1, SOCS3 |
| Chemical carcinogenesis - reactive oxygen species | 1.78 | 10 | 4.90E-08 | 1.70E-05 | GSTM3, MGST2, NDUFV3, SOD2, PPIF, CAT, EGFR, MET, NRAS, MAPK1, MAPK8, JUN, PIK3CD, RAC1, ABL2, NFE2L2, HMOX1 |
| Non-small cell lung cancer | 3.30 | 10 | 6.40E-08 | 7.00E-06 | CDK4, PIK3CD, EGFR, MET, NRAS, MAPK1, TP53, BAX, KIF5B, RET, EML4 |
| Hepatitis C | 2.53 | 10 | 7.10E-08 | 7.10E-08 | LDLR, IFNA1, SOCS3, IFNAR2, JAK1, PPP2R1B, PPP2R2A, EGFR, NRAS, YWHAZ, YWHAE, MAPK1, PIK3CD, CTNNB1, TP53, CDK4, CASP3, BAX |
| Prostate cancer | 2.01 | 10 | 7.10E-08 | 7.10E-08 | CCNE2, EGFR, IGF1R, PIK3CD, TP53, CREB1, CTNNB1, NRAS, MAPK1 |
| Cellular senescence | 2.93 | 10 | 7.10E-08 | 7.10E-08 | TGFBR1, CDK4, RBL1, PIK3CD, CCNE2, NRAS, CCNA2, MYBL2, LIN54, TP53, PPP1CC, MAPK1, MAP2K3, ZFP36L1, ZFP36L2, CAPN2, CALM3, PPP3CA, PPP3CB, PPP3R1, PPID |
| Th17 cell differentiation | 2.00 | 10 | 7.80E-08 | 1.80E-03 | TGFBR1, JAK1, HLA-DQA1, PPP3CA, PPP3CB, PPP3R1, MAPK1, MAPK8, JUN, RUNX1 |
| Neurotrophin signaling pathway | 2.75 | 10 | 8.30E-08 | 4.90E-07 | BDNF, NRAS, MAPK1, KIDINS220, FRS2, SHC1, GAB1, PIK3CD, CALM3, RAC1, MAPK8, JUN, TP53, BAX, YWHAE |
| mRNA surveillance pathway | 2.76 | 10 | 1.40E-07 | 3.90E-05 | NXT1, TARDBP, NUDT21, CPSF1, CPSF3, CPSF4, WDR82, PPP1CC, PABPC1, PPP2R1B, PPP2R2A, PPP2R5C |
| GnRH signaling pathway | 1.86 | 10 | 1.40E-07 | 5.00E-05 | CALM3, MAP2K3, PLA2G4A, MAPK8, JUN, EGFR, NRAS, MAPK1 |
| Estrogen signaling pathway | 2.21 | 10 | 1.60E-07 | 1.00E-06 | NCOA3, KRT10, KRT14, KRT18, JUN, SP1, CREB1, EGFR, SHC1, NRAS, MAPK1, PIK3CD, GNAI2, CALM3 |
| ErbB signaling pathway | 2.60 | 10 | 1.60E-07 | 2.10E-06 | EGFR, ABL2, PAK1, MAPK8, JUN, SHC1, NRAS, MAPK1, GAB1, PIK3CD |
| Focal adhesion | 2.29 | 10 | 2.00E-07 | 4.10E-07 | LAMC1, THBS1, THBS2, ITGA2, ITGA6, ITGB4, IGF1R, EGFR, MET, PPP1CC, PPP1R12A, CAPN2, CTNNB1, PIK3CD, VAV3, RAC1, PAK1, MAPK8, JUN, SHC1, MAPK1 |
| Bacterial invasion of epithelial cells | 2.82 | 10 | 2.00E-07 | 8.40E-04 | CTNNB1, MET, GAB1, PIK3CD, RAC1, WASF1, ARPC5, SHC1, CD2AP |
| Melanoma | 2.70 | 10 | 2.00E-07 | 3.60E-07 | MET, IGF1R, EGFR, NRAS, MAPK1, CDK4, PIK3CD, TP53, BAX |
| FoxO signaling pathway | 2.70 | 10 | 2.20E-07 | 7.20E-07 | TGFBR1, PRKAA1, NLK, SKP2, MAPK8, IGF1R, PIK3CD, EGFR, NRAS, MAPK1, PRMT1, PLK2, BNIP3, GABARAPL2, CAT, SOD2 |
| T cell receptor signaling pathway | 2.35 | 10 | 2.20E-07 | 2.00E-06 | VAV3, PAK1, PPP3CA, PPP3CB, PPP3R1, NRAS, MAPK1, JUN, MAPK8, PIK3CD, CDK4 |
| Glioma | 2.88 | 10 | 2.30E-07 | 4.20E-07 | EGFR, IGF1R, CALM3, SHC1, NRAS, MAPK1, PIK3CD, TP53, CDK4, BAX |
| Thyroid cancer | 4.09 | 10 | 2.50E-07 | 3.20E-05 | RET, NRAS, MAPK1, PPARG, TP53, BAX, CTNNB1 |
| Chronic myeloid leukemia | 2.84 | 10 | 2.90E-07 | 8.20E-06 | PIK3CD, BCL2L1, TP53, NRAS, MAPK1, SHC1, CDK4, BAX, TGFBR1, RUNX1 |
| Bladder cancer | 3.16 | 10 | 3.60E-07 | 1.90E-05 | NRAS, MAPK1, TP53, CDK4, THBS1, EGFR |
| Toxoplasmosis | 2.51 | 10 | 3.90E-07 | 6.30E-04 | JAK1, HLA-DQA1, SOCS1, MAPK1, MAPK8, MAP2K3, BCL2L1, LAMC1, ITGA6, LDLR, CASP3, GNAI2, PPIF |
| Transcriptional misregulation in cancer | 2.14 | 10 | 4.40E-07 | 2.80E-06 | RUNX1, PER2, JUP, CCNA2, CCNT1, CCNT2, RUNX2, HOXA10, IGF1R, BCL2L1, PPARG, TP53, SP1, PAX7, PAX3, MET, BAX |
| Small cell lung cancer | 2.58 | 10 | 5.70E-07 | 1.90E-05 | TP53, BAX, CASP3, CDK4, SKP2, CCNE2, LAMC1, ITGA2, ITGA6, PIK3CD, BCL2L1 |
| Apoptosis | 2.40 | 10 | 9.60E-07 | 9.70E-05 | TNFRSF10B, CASP3, CASP7, BAX, LMNB2, CAPN2, CTSL, BCL2L1, MAPK8, JUN, TP53, PMAIP1, PIK3CD, NRAS, MAPK1 |
| Renal cell carcinoma | 2.99 | 10 | 9.70E-07 | 1.00E-06 | VHL, MET, GAB1, PIK3CD, NRAS, MAPK1, JUN, RAC1, PAK1 |
| Measles | 2.04 | 10 | 1.00E-06 | 1.00E-04 | IFNA1, MAPK8, JUN, IFNAR2, JAK1, ADAR, TP53, BAX, BCL2L1, CASP3, CCNE2, CDK4, PIK3CD |
| Regulation of actin cytoskeleton | 2.02 | 10 | 1.20E-06 | 3.60E-05 | EGFR, ITGA2, ITGA6, ITGB4, GNA13, GNG12, NRAS, PIK3CD, VAV3, MAPK1, RAC1, PAK1, PPP1CC, PPP1R12A, SLC9A1, PIP4K2A, NCKAP1, WASF1, ARPC5, SPATA13 |
| Apoptosis - multiple species | 5.04 | 10 | 1.20E-06 | 2.20E-04 | CASP7, CASP3, PMAIP1, BCL2L1, BAX, MAPK8, BECN1 |
| Salmonella infection | 2.45 | 10 | 1.20E-06 | 1.60E-05 | VPS33A, DYNC1H1, DYNC1LI1, DYNC2LI1, DYNLL2, DCTN4, DCTN5, TUBB3, FYCO1, KIF5B, RAC1, NCKAP1, ARPC5, PAK1, PIK3CD, ARF6, KPNA1, KPNA4, MAPK1, MAP2K3, MAPK8, JUN, CTNNB1, TNFRSF10B, BAX, CASP3, CASP7, RHOB |
| Endometrial cancer | 2.98 | 10 | 1.40E-06 | 3.40E-06 | EGFR, PIK3CD, NRAS, MAPK1, MLH1, CTNNB1, TP53, BAX |
| Pertussis | 1.99 | 10 | 1.50E-06 | 4.60E-03 | CALM3, CASP3, CASP7, MAPK8, MAPK1, JUN, GNAI2 |
| Natural killer cell mediated cytotoxicity | 2.40 | 10 | 1.50E-06 | 6.50E-04 | VAV3, RAC1, PAK1, MAPK1, PIK3CD, SHC1, NRAS, PPP3CA, PPP3CB, PPP3R1, IFNA1, IFNAR2, TNFRSF10B, CASP3 |
| Arrhythmogenic right ventricular cardiomyopathy | 2.56 | 10 | 1.90E-06 | 4.10E-04 | ITGA2, ITGA6, ITGB4, DAG1, CTNNB1, ATP2A2, CDH2, JUP, DSG2 |
| Insulin signaling pathway | 2.23 | 10 | 2.10E-06 | 7.50E-06 | PIK3CD, PPP1CC, PHKA1, CALM3, FASN, HK1, PRKAA1, SHC1, NRAS, MAPK1, MKNK2, SOCS1, SOCS3, MAPK8 |
| AMPK signaling pathway | 2.75 | 10 | 2.40E-06 | 4.80E-05 | CAB39, PRKAA1, PFKFB2, CREB1, CCNA2, EEF2, FASN, SCD, PPP2R1B, PPP2R2A, PPP2R5C, RAB14, IGF1R, PIK3CD, PPARG |
| IL-17 signaling pathway | 1.90 | 10 | 2.50E-06 | 8.50E-03 | CASP3, JUN, ANAPC5, MAPK8, MAPK1, USP25, SRSF1, MMP13 |
| Vasopressin-regulated water reabsorption | 3.44 | 10 | 2.70E-06 | 5.20E-03 | CREB1, DYNC1H1, DYNC1LI1, DYNC2LI1, DYNLL2, DCTN4, DCTN5 |
| Influenza A | 2.42 | 10 | 2.70E-06 | 3.20E-04 | DNAJB1, DNAJC3, IFNA1, IFNAR2, JAK1, SOCS3, ADAR, HLA-DQA1, PIK3CD, CDK4, TNFRSF10B, BAX, CASP3, CPSF4, NXT1, MAPK1, XPO1, KPNA1, KPNA6 |
| HIF-1 signaling pathway | 1.82 | 10 | 2.80E-06 | 2.80E-06 | EGFR, IGF1R, MAPK1, MKNK2, PIK3CD, VHL, HMOX1, HK1, ALDOA |
| PD-L1 expression and PD-1 checkpoint pathway in cancer | 2.91 | 10 | 2.80E-06 | 1.70E-05 | EGFR, NRAS, MAPK1, JUN, EML4, PIK3CD, JAK1, BATF2, MAP2K3, PPP3CA, PPP3CB, PPP3R1 |
| NOD-like receptor signaling pathway | 1.48 | 10 | 3.00E-06 | 3.50E-03 | MAPK1, MAPK8, JUN, GABARAPL2, MAP1LC3B, IFNA1, BCL2L1, DHX33, TXNIP, YWHAE, IFNAR2, JAK1 |
| Prolactin signaling pathway | 2.47 | 10 | 3.90E-06 | 2.70E-04 | SHC1, PIK3CD, NRAS, MAPK1, MAPK8, SOCS1, SOCS3, SOCS6 |
| C-type lectin receptor signaling pathway | 2.14 | 10 | 4.70E-06 | 2.80E-05 | NRAS, CALM3, PPP3CA, PPP3CB, PPP3R1, MAPK1, MAPK8, JUN, PAK1, PIK3CD |
| Oocyte meiosis | 2.91 | 10 | 4.70E-06 | 4.70E-06 | IGF1R, CPEB4, MAPK1, YWHAZ, YWHAE, PPP1CC, CUL1, ANAPC5, CDC20, CCNE2, PPP2R5C, PPP2R1B, CALM3, PPP3CA, PPP3CB, PPP3R1 |
| Chagas disease | 1.91 | 10 | 4.80E-06 | 3.60E-03 | MAPK8, MAPK1, JUN, PPP2R1B, PPP2R2A, CALR, GNAI2, PIK3CD, TGFBR1 |
| Pathogenic Escherichia coli infection | 2.34 | 10 | 4.80E-06 | 8.10E-06 | RAC1, ARF6, NCKAP1, WASF1, ARPC5, MYO10, GNA13, TUBB3, NCL, MAPK1, MAPK8, JUN, TNFRSF10B, TMBIM6, BAX, CASP3, CASP7, PAK1, SEC24B, SEC24A, TMED10 |
| Non-alcoholic fatty liver disease | 1.85 | 10 | 4.80E-06 | 2.40E-03 | SOCS3, PIK3CD, MLXIP, PRKAA1, PPARG, RAC1, MAPK8, ITCH, JUN, BAX, CASP3, CASP7, NDUFV3 |
| Toll-like receptor signaling pathway | 1.66 | 10 | 5.00E-06 | 4.80E-04 | RAC1, PIK3CD, MAPK1, MAP2K3, MAPK8, JUN, IFNA1, IFNAR2 |
| Thyroid hormone signaling pathway | 2.32 | 10 | 5.00E-06 | 5.00E-06 | NRAS, MAPK1, TP53, NCOA3, MED13, MED14, ATP2A2, CTNNB1, NOTCH2, SLC9A1, PIK3CD, TBC1D4, PFKFB2 |
| Parathyroid hormone synthesis, secretion and action | 1.85 | 10 | 5.10E-06 | 6.80E-04 | GNAI2, SP1, MAPK1, MEF2D, CREB1, RUNX2, MMP13, GNA13, EGFR |
| Phospholipase D signaling pathway | 1.34 | 10 | 5.40E-06 | 1.20E-03 | EGFR, GAB1, SHC1, NRAS, PIK3CD, ARF6, PLA2G4A, MAPK1, GNA13 |
| Nucleocytoplasmic transport | 2.29 | 10 | 5.40E-06 | 5.00E-05 | NUP133, KPNA1, KPNA4, KPNA6, TNPO1, TNPO3, XPO1, XPOT, XPO7, EEF1A1, NXT1 |
| Fc epsilon RI signaling pathway | 2.54 | 10 | 5.80E-06 | 5.80E-06 | PIK3CD, RAC1, MAP2K3, MAPK8, VAV3, NRAS, MAPK1, PLA2G4A |
| cAMP signaling pathway | 1.63 | 10 | 6.60E-06 | 6.60E-06 | EDNRA, GNAI2, CALM3, MAPK8, VAV3, RAC1, PAK1, PIK3CD, MAPK1, PPP1CC, CREB1, BDNF, JUN, PPP1R12A, ATP2A2, SLC9A1 |
| Autophagy - animal | 2.18 | 10 | 7.60E-06 | 8.00E-06 | IGF1R, PIK3CD, NRAS, MAPK1, BNIP3, PRKAA1, MAPK8, BCL2L1, BECN1, MTMR3, GABARAPL2, MAP1LC3B, LAMP1, CTSL |
| Fluid shear stress and atherosclerosis | 2.19 | 10 | 7.80E-06 | 7.80E-06 | CALM3, NFE2L2, HMOX1, GSTM3, MGST2, CTNNB1, PRKAA1, PIK3CD, MAPK8, JUN, CTSL, BMPR2, RAC1, TP53 |
| Cushing syndrome | 1.68 | 10 | 8.80E-06 | 4.90E-05 | CREB1, RBBP5, CDK4, GNAI2, CCNE2, MAPK1, EGFR, SP1, NCEH1, LDLR, WNT5A, CTNNB1 |
| Growth hormone synthesis, secretion and action | 1.82 | 10 | 8.90E-06 | 5.90E-05 | GNAI2, CREB1, SHC1, NRAS, MAPK1, PIK3CD, SOCS1, SOCS3, MAP2K3, MAPK8 |
| Axon guidance | 1.94 | 10 | 1.00E-05 | 3.00E-04 | PPP3CA, PPP3CB, PPP3R1, RAC1, ABLIM1, PAK1, NRAS, MAPK1, GNAI2, PLXNA1, SEMA6A, SEMA6D, MET, WNT5A, PIK3CD, BMPR2 |
| Sphingolipid signaling pathway | 2.77 | 10 | 1.00E-05 | 5.50E-05 | SPTLC1, SPTLC3, BAX, MAPK8, PPP2R1B, PPP2R2A, PPP2R5C, TP53, PIK3CD, GNAI2, MAPK1, SGPL1, NRAS, RAC1, GNA13 |
| Coronavirus disease - COVID-19 | 1.13 | 10 | 1.30E-05 | 5.10E-03 | EGFR, JAK1, RPS15A, RPS24, RPL19, PIK3CD, MAPK1, MAPK8, JUN, IFNA1, IFNAR2, ADAR |
| Yersinia infection | 1.44 | 10 | 1.60E-05 | 1.20E-03 | RAC1, ARF6, ARPC5, MAPK1, MAP2K3, MAPK8, JUN, PIK3CD, VAV3 |
| Long-term depression | 2.61 | 10 | 1.70E-05 | 6.00E-03 | PPP2R1B, NRAS, MAPK1, GNAI2, GNA13, PLA2G4A, IGF1R |
| Longevity regulating pathway | 2.43 | 10 | 1.70E-05 | 1.70E-05 | IGF1R, NRAS, PIK3CD, CREB1, SOD2, CAT, PRKAA1, BAX, TP53, PPARG |
| Wnt signaling pathway | 1.59 | 10 | 1.70E-05 | 1.80E-05 | WNT5A, CTNNB1, NLK, JUN, TP53, TBL1XR1, CUL1, RAC1, MAPK8, PPP3CA, PPP3CB, PPP3R1 |
| Signaling pathways regulating pluripotency of stem cells | 2.18 | 10 | 1.90E-05 | 1.10E-04 | LIFR, JAK1, SOX2, MAPK1, PIK3CD, NODAL, BMPR2, ID4, WNT5A, CTNNB1, NRAS, IGF1R, RIF1, ZFHX3 |
| Tight junction | 1.61 | 10 | 2.40E-05 | 5.60E-04 | RAC1, PPP2R1B, PPP2R2A, NEDD4L, MAPK8, JUN, CDK4, YBX3, PCNA, RUNX1, ARPC5, PRKAA1 |
| B cell receptor signaling pathway | 2.74 | 10 | 2.60E-05 | 1.80E-04 | VAV3, RAC1, PPP3CA, PPP3CB, PPP3R1, RASGRP3, NRAS, MAPK1, JUN, PIK3CD |
| Oxytocin signaling pathway | 2.02 | 10 | 3.10E-05 | 3.50E-04 | NRAS, MAPK1, PLA2G4A, JUN, EEF2, CALM3, PPP3CA, PPP3CB, PPP3R1, PRKAA1, PPP1CC, PPP1R12A, GNAI2, EGFR |
| Insulin resistance | 2.04 | 10 | 3.50E-05 | 3.50E-05 | PPP1CC, PIK3CD, SOCS3, MAPK8, TBC1D4, PRKAA1, PTPA, PPARGC1B, MLXIP, CREB1 |
| Diabetic cardiomyopathy | 1.27 | 10 | 3.60E-05 | 3.60E-05 | ATP2A2, SP1, RAC1, MAPK8, PTPA, PPP1CC, PIK3CD, TBC1D4, NDUFV3, PPIF, TGFBR1 |
| cGMP-PKG signaling pathway | 1.88 | 10 | 3.70E-05 | 3.70E-05 | EDNRA, GNA13, PPP3CA, PPP3CB, PPP3R1, MEF2D, ATP2A2, PPP1R12A, PPP1CC, CALM3, GNAI2, PPIF, CREB1, MAPK1 |
| Leishmaniasis | 1.40 | 10 | 4.10E-05 | 5.10E-03 | MAPK1, JUN, JAK1, HLA-DQA1, EEF1A1 |
| Parkinson disease | 1.48 | 10 | 4.30E-05 | 1.40E-04 | GNAI2, UBA1, UBE2L3, CALM3, HSPA5, SLC11A2, NDUFV3, PINK1, PPIF, CASP3, BCL2L1, BAX, KIF5B, TUBB3, TP53, NFE2L2, MAPK8 |
| Ribosome biogenesis in eukaryotes | 1.99 | 10 | 4.40E-05 | 4.40E-03 | UTP15, WDR75, UTP6, UTP14A, BMS1, RPP40, GNL3L, XPO1, NXT1 |
| Type II diabetes mellitus | 3.29 | 10 | 4.60E-05 | 1.70E-03 | PIK3CD, MAPK1, SOCS1, SOCS3, MAPK8, HK1, PKM |
| Nucleotide excision repair | 2.35 | 10 | 5.00E-05 | 9.40E-03 | RAD23B, GTF2H1, GTF2H2C, RPA2, PCNA |
| Gap junction | 1.26 | 10 | 5.10E-05 | 2.90E-03 | GNAI2, EGFR, NRAS, MAPK1, TUBB3 |
| Tuberculosis | 1.57 | 10 | 5.40E-05 | 3.00E-03 | CASP3, BAX, JAK1, CREB1, HLA-DQA1, MAPK1, MAPK8, IFNA1, CALM3, LAMP1, PPP3CA, PPP3CB, PPP3R1 |
| VEGF signaling pathway | 2.93 | 10 | 5.90E-05 | 6.90E-05 | NRAS, MAPK1, PLA2G4A, PPP3CA, PPP3CB, PPP3R1, PIK3CD, RAC1 |
| Chemical carcinogenesis - receptor activation | 1.36 | 10 | 6.10E-05 | 3.90E-04 | PIK3CD, NRAS, MAPK1, JUN, CREB1, GNAI2, EGFR, NOTCH2, KPNA1, KPNA4, KPNA6, GSTM3, MGST2 |
| Th1 and Th2 cell differentiation | 2.14 | 10 | 7.50E-05 | 8.20E-03 | JAK1, HLA-DQA1, PPP3CA, PPP3CB, PPP3R1, MAPK1, MAPK8, JUN, NOTCH2 |
| Alcoholism | 2.45 | 10 | 8.60E-05 | 3.20E-04 | GNAI2, GNG2, GNG5, GNG12, ADORA2B, CREB1, BDNF, SHC1, NRAS, MAPK1, CALM3, PPP1CC |
| Dopaminergic synapse | 2.29 | 10 | 8.80E-05 | 8.80E-05 | CALM3, PPP3CA, PPP3CB, CREB1, MAPK8, PPP1CC, GNAI2, GNG2, GNG5, GNG12, PPP2R1B, PPP2R2A, PPP2R5C, KIF5B |
| Chemokine signaling pathway | 1.26 | 10 | 9.70E-05 | 3.70E-03 | GNAI2, SHC1, NRAS, MAPK1, PIK3CD, VAV3, RAC1, PAK1, GNG2, GNG5, GNG12 |
| Hippo signaling pathway | 1.43 | 10 | 1.10E-04 | 1.10E-04 | PPP2R1B, PPP2R2A, PPP1CC, TGFBR1, BMPR2, WNT5A, YWHAZ, YWHAE, CTNNB1, SOX2 |
| Ferroptosis | 3.69 | 10 | 1.20E-04 | 8.70E-03 | SLC7A11, GCLC, ACSL4, TP53, SLC11A2, MAP1LC3B, HMOX1 |
| Protein export | 4.91 | 10 | 1.20E-04 | 2.50E-04 | SEC62, HSPA5, SPCS3, SEC11C, SEC11A |
| Phagosome | 2.28 | 10 | 1.30E-04 | 1.60E-03 | VAMP3, STX7, HLA-DQA1, ATP6V1B2, ATP6V1C1, DYNC1H1, DYNC1LI1, TUBB3, LAMP1, CTSL, CALR, ITGA2, THBS1, THBS2, OLR1, RAC1 |
| SNARE interactions in vesicular transport | 3.28 | 10 | 1.70E-04 | 8.80E-04 | STX7, STX16, USE1, VAMP3, YKT6 |
| Acute myeloid leukemia | 2.26 | 10 | 2.00E-04 | 1.50E-03 | PIK3CD, NRAS, MAPK1, RUNX1, PER2, CCNA2, JUP |
| Long-term potentiation | 2.26 | 10 | 2.40E-04 | 1.30E-03 | PPP1CC, PPP3CA, PPP3CB, PPP3R1, CALM3, NRAS, MAPK1 |
| Prion disease | 1.35 | 10 | 2.50E-04 | 1.50E-03 | LAMC1, BAX, HSPA5, PPP3CA, PPP3CB, PPP3R1, PPIF, CASP3, NDUFV3, PIK3CD, RAC1, MAPK8, MAPK1, CREB1, KIF5B, TUBB3 |
| Necroptosis | 1.46 | 10 | 3.10E-04 | 3.40E-02 | PPID, MAPK8, PLA2G4A, CAPN2, TNFRSF10B, IFNA1, IFNAR2, JAK1, BAX |
| Apelin signaling pathway | 2.24 | 10 | 3.30E-04 | 1.20E-03 | GNAI2, GNG2, GNG5, GNG12, CALM3, SLC9A1, NRAS, MAPK1, BECN1, MAP1LC3B, PRKAA1, GNA13, MEF2D, TGFBR1 |

From left to right, the table reports the description of the enriched term, the fold enrichment value for the enriched term, the number of iterations that the given term was found to enriched over all iterations (occurrence), the lowest adjusted-*p* value of the given term over all iterations, the highest adjusted-*P* value of the given term over all iterations, and the list of miRNAs target genes.

**Supplementary Results Figure 1** Gene Ontology terms Tree of down-regulated miRNAs target genes. Pathways with many shared genes are clustered together. Bigger dots indicate more significant *p* values.


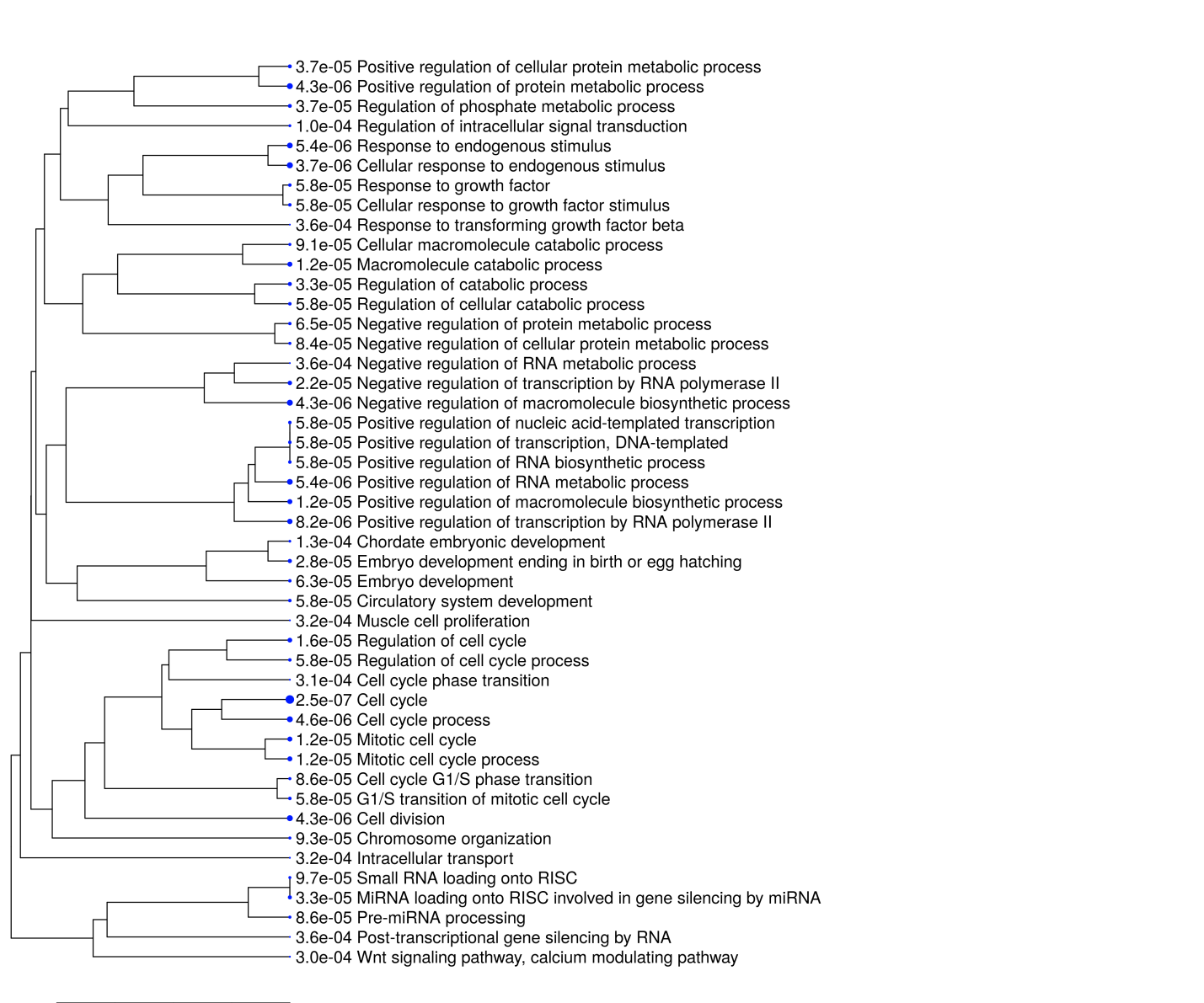


**Supplementary Results Figure 2** Gene Ontology terms Tree of up-regulated miRNAs target genes. Pathways with many shared genes are clustered together. Bigger dots indicate more significant *p* values.


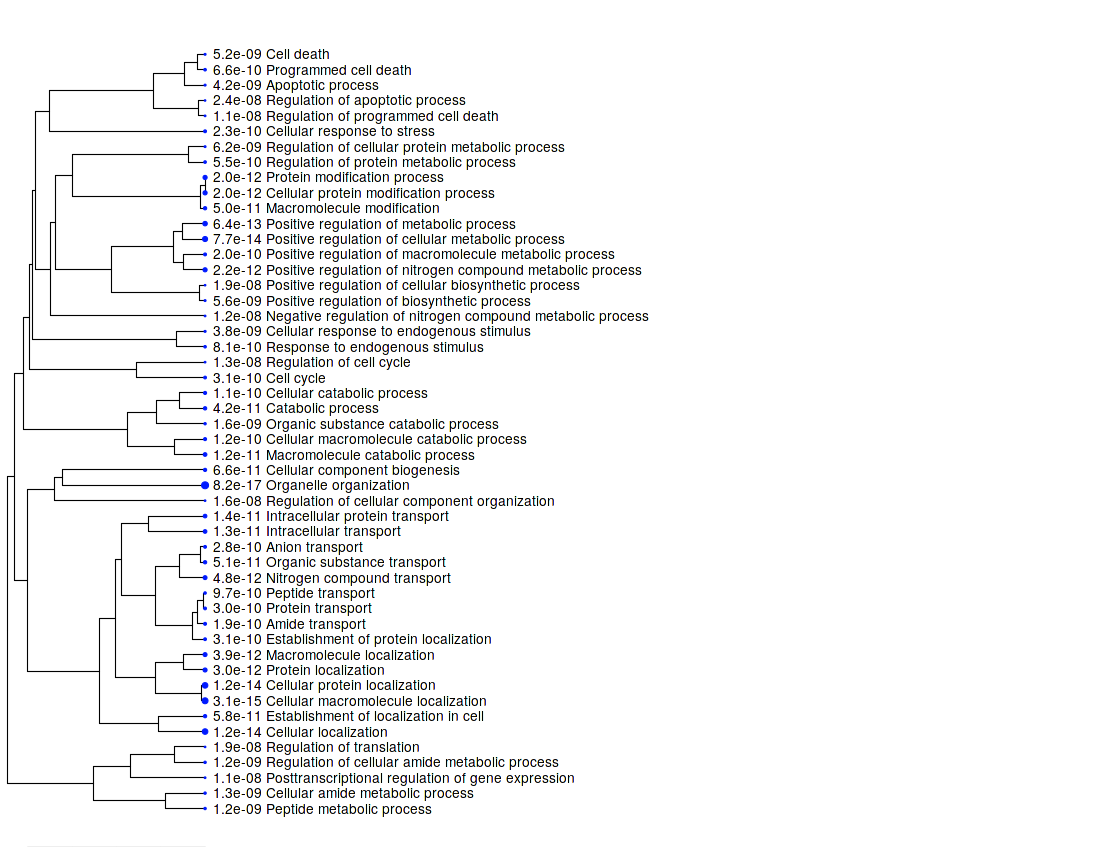


**Supplementary References**

1. Skovronova R, Grange C, Dimuccio V, Deregibus MC, Camussi G, Bussolati B. Surface Marker Expression in Small and Medium/Large Mesenchymal Stromal Cell-Derived Extracellular Vesicles in Naive or Apoptotic Condition Using Orthogonal Techniques. Cells 2021;10:2948.
2. Bugianesi E, Gastaldelli A, Vanni E, et al. Insulin resistance in non-diabetic patients with non-alcoholic fatty liver disease: sites and mechanisms. Diabetologia 2005;48:634–642.
3. Gastaldelli A, Casolaro A, Pettiti M, Nannipieri M, Ciociaro D, Frascerra S, et al. Effect of pioglitazone on the metabolic and hormonal response to a mixed meal in type II diabetes. Clin Pharmacol Ther 2007;81:205–212.
4. Kleiner DE, Brunt EM, Van Natta M, et al. Design and validation of a histological scoring system for nonalcoholic fatty liver disease. Hepatology 2005;41:1313–1321.
5. European Association for the Study of the Liver (EASL); European Association for the Study of Diabetes (EASD); European Association for the Study of Obesity (EASO). EASL-EASD-EASO Clinical Practice Guidelines for the management of non-alcoholic fatty liver disease. J Hepatol 2016;64:1388–1402.
6. Newsome PN, Sasso M, Deeks JJ, et al. FibroScan-AST (FAST) score for the non-invasive identification of patients with non-alcoholic steatohepatitis with significant activity and fibrosis: a prospective derivation and global validation study. Lancet Gastroenterol Hepatol 2020;5:362–373.
7. Ru Y, Kechris KJ, Tabakoff B, et al. The multiMiR R package and database: integration of microRNA-target interactions along with their disease and drug associations. Nucleic Acids Res 2014;42:e133.
8. Ge SX, Jung D, Yao R. ShinyGO: a graphical gene-set enrichment tool for animals and plants. Bioinformatics 2020;36:2628–2629.
9. Ulgen E, Ozisik O, Sezerman OU. pathfindR: An R Package for Comprehensive Identification of Enriched Pathways in Omics Data Through Active Subnetworks. Front Genet 2019;10:858.
10. Martin M. Cutadapt removes adapter sequences from high-throughput sequencing reads. EMBnet J 2011;17:10.
11. Li H. Aligning sequence reads, clone sequences, and assembly contigs with BWA-MEM. 2012; arXiv:1303.3997v2 [q-bio.GN].
12. Love MI, Huber W, Anders S. Moderated estimation of fold change and dispersion for RNA-seq data with DESeq2. Genome Biol 2014;15:550.

**TABLE S1** Differentially expressed miRNAs in serum EVs according to the diagnosis of steatohepatitis.

| **miRNAs** | **log_2_ FC** | **log_2_ FC SE** | ***p* value** | **Adjusted *p* value*** |
| --- | --- | --- | --- | --- |
| miR-30a-5p | 0.32 | 0.09 | <0.001 | 0.029 |
| miR-130b-5p | -0.37 | 0.11 | 0.001 | 0.029 |
| miR-143-3p | 0.38 | 0.11 | <0.001 | 0.029 |
| let-7i-5p | -0.17 | 0.05 | 0.001 | 0.029 |
| miR-1301-3p | -0.29 | 0.09 | 0.001 | 0.029 |
| miR-193a-5p | 0.46 | 0.14 | 0.001 | 0.029 |
| miR-148a-3p | 0.31 | 0.09 | 0.001 | 0.029 |
| miR-340-3p | -0.25 | 0.08 | 0.002 | 0.034 |
| miR-27b-3p | 0.22 | 0.07 | 0.002 | 0.034 |
| miR-100-5p | 0.57 | 0.19 | 0.002 | 0.034 |
| miR-375-3p | 0.48 | 0.15 | 0.002 | 0.034 |
| miR-423-5p | -0.36 | 0.12 | 0.002 | 0.035 |
| let-7d-5p | -0.20 | 0.06 | 0.003 | 0.040 |
| miR-151a-3p | -0.15 | 0.05 | 0.004 | 0.041 |
| miR-215-5p | 0.37 | 0.13 | 0.004 | 0.041 |
| miR-29a-3p | 0.21 | 0.07 | 0.003 | 0.041 |
| miR-128-3p | -0.12 | 0.04 | 0.004 | 0.041 |
| let-7f-5p | -0.16 | 0.06 | 0.005 | 0.043 |
| miR-192-5p | 0.36 | 0.13 | 0.005 | 0.043 |
| miR-122-5p | 0.54 | 0.19 | 0.005 | 0.046 |
| miR-26a-5p | -0.15 | 0.05 | 0.006 | 0.046 |
| miR-338-5p | 0.33 | 0.12 | 0.006 | 0.047 |
| miR-103a-3p | -0.13 | 0.05 | 0.006 | 0.047 |

*adjusted for library, age, gender, BMI, and T2DM. Abbreviations: fold change (FC), miRNA (microRNA), standard error (SE).

**TABLE S2** Differentially expressed miRNAs in serum EVs according to the presence of significant fibrosis (F ≥ 2).

| **miRNAs** | **log_2_ FC** | **log_2_ FC SE** | ***p* value** | **Adjusted *p* value*** |
| --- | --- | --- | --- | --- |
| miR-221-3p | -0.24 | 0.06 | <0.001 | 0.010 |
| miR-378c | 0.38 | 0.10 | <0.001 | 0.010 |
| miR-378a-3p | 0.33 | 0.09 | <0.001 | 0.010 |
| miR-146a-5p | -0.21 | 0.06 | <0.001 | 0.010 |
| miR-30a-5p | 0.31 | 0.09 | <0.001 | 0.010 |
| miR-122-5p | 0.67 | 0.19 | <0.001 | 0.012 |
| miR-1246 | 0.73 | 0.21 | <0.001 | 0.012 |
| miR-27b-3p | 0.24 | 0.07 | 0.001 | 0.014 |
| miR-99a-5p | 0.42 | 0.12 | 0.001 | 0.016 |
| miR-28-5p | -0.23 | 0.07 | 0.001 | 0.016 |
| miR-375-3p | 0.47 | 0.15 | 0.002 | 0.026 |
| miR-30c-5p | -0.18 | 0.06 | 0.003 | 0.029 |
| miR-103a-3p | -0.15 | 0.05 | 0.002 | 0.029 |
| miR-191-5p | -0.15 | 0.05 | 0.003 | 0.029 |
| let-7d-5p | -0.17 | 0.06 | 0.003 | 0.029 |
| miR-199a-5p | -0.25 | 0.08 | 0.002 | 0.029 |
| let-7b-3p | 0.31 | 0.10 | 0.003 | 0.029 |
| let-7f-5p | -0.17 | 0.05 | 0.003 | 0.029 |
| miR-374b-5p | -0.27 | 0.09 | 0.003 | 0.029 |
| miR-199a-3p | -0.23 | 0.08 | 0.004 | 0.032 |
| miR-101-3p | 0.38 | 0.13 | 0.004 | 0.032 |
| miR-151a-5p | -0.26 | 0.09 | 0.004 | 0.034 |
| miR-340-5p | -0.15 | 0.06 | 0.006 | 0.041 |
| miR-584-5p | -0.17 | 0.06 | 0.005 | 0.041 |
| miR-185-5p | 0.31 | 0.12 | 0.007 | 0.043 |
| miR-532-5p | 0.24 | 0.09 | 0.007 | 0.043 |
| miR-4443 | 0.38 | 0.14 | 0.007 | 0.043 |
| miR-98-5p | -0.18 | 0.07 | 0.007 | 0.043 |
| miR-1301-3p | -0.24 | 0.09 | 0.007 | 0.043 |
| miR-486-3p | 0.35 | 0.13 | 0.008 | 0.046 |
| miR-320c | 0.29 | 0.11 | 0.008 | 0.047 |

*adjusted for library, age, gender, BMI, and T2DM. Abbreviations: fold change (FC), miRNA (microRNA), standard error (SE).

**TABLE S3 Characteristics of the patients that underwent fluxomic studies at the time of liver biopsy.**

| **Variables** | **Total, *n* = 54** |
| --- | --- |
| Age (years), median (IQR) | 43.5 (33.0–52.0) |
| Gender (M/F) | 42/12 |
| BMI (kg/m^2^), median (IQR) | 27.2 (25.0–30.1) |
| Waist (cm), median (IQR) | 95 (89–102) |
| Hypertension, *n* (%) | 10 (18.5%) |
| ALT (U/L), median (IQR) | 62 (41–88) |
| AST (U/L), median (IQR) | 34 (26–48) |
| γGT (U/L), median (IQR) | 46 (28–106) |
| Platelets (x10^9^/L), median (IQR) | 226 (190–279) |
| Albumin (g/dL), median (IQR) | 4.4 (4.1–4.6) |
| Total bilirubin (mg/dL), median (IQR) | 0.8 (0.5–1.0) |
| Liver Histology |  |
| Steatohepatitis, *n* (%) | 39 (72.2%) |
| Liver fibrosis, *n* (%)  F0  F1  F2  F3  F4 | 18 (33.3%)  5 (9.3%)  18 (33.3%)  11 (20.4%)  2 (3.7%) |
| “At risk MASH” | 17 (31.5%) |
| Glucose metabolism  Fasting glucose (mg/dL), median (IQR)  Fasting insulin (IU/L), median (IQR)  HOMA-IR, median (IQR)  Hep-IR (µmol/min/kg * IU/mL), median (IQR) | 93 (89–104)  11.7 (8.9–16.6)  2.76 (2.07–3.68)  115 (86–142) |
| Lipid metabolism  Total cholesterol (mg/dL), median (IQR)  HDL cholesterol (mg/dL), median (IQR)  Triglycerides (mg/dL), median (IQR)  Lipolysis (µmol * min^-1^), median (IQR)  FFAs (mmol/L), median (IQR)  AT-IR (mmol/L * IU/mL), median (IQR)  SFAs (µmol), median (IQR)  MUFAs (µmol), median (IQR)  PUFAs (µmol), median (IQR) | 190 (175–209)  47 (38–53)  104 (83–165)  2.53 (1.99–3.30)  0.63 (0.46–0.76)  7.1 (4.7–10.9)  174 (130–256)*  163 (117–228)*  148 (104–171)* |

*available in a subgroup of 39 patients. Abbreviations: adipose tissue insulin resistance by free fatty acids (AT-IR), alanine aminotransferase (ALT), aspartate aminotransferase (AST), body mass index (BMI), gamma-glutamyl transferase (γGT), free fatty acids (FFAs), hepatic insulin resistance (Hep-IR), high-density lipoprotein (HDL), homeostasis model assessment of insulin resistance (HOMA-IR), interquartile range (IQR), monounsaturated fatty acids (MUFAs), number (*n*), metabolic dysfunction-associated steatohepatitis (MASH), polyunsaturated fatty acids (PUFAs), saturated fatty acids (SFA).

**TABLE S4** Correlations between circulating miRNAs with lipolysis, free fatty acids and adipose tissue insulin resistance index.

|  | **Glycerol Ra** | | **FFAs** | | **AT-IR** | |
| --- | --- | --- | --- | --- | --- | --- |
| **Variables** | ***r*** | ***p* value** | ***r*** | ***p* value** | ***r*** | ***p* value** |
| miR-122-5p | 0.03 | 0.813 | 0.35 | 0.009 | 0.35 | 0.009 |
| miR-375-3p | 0.19 | 0.159 | 0.42 | 0.001 | 0.36 | 0.008 |
| let-7f-5p | -0.35 | 0.010 | -0.45 | <0.001 | -0.45 | <0.001 |
| let-7d-5p | -0.30 | 0.025 | -0.31 | 0.021 | -0.29 | 0.034 |
| miR-27b | 0.07 | 0.632 | 0.25 | 0.107 | 0.28 | 0.063 |
| miR-30a | -0.10 | 0.576 | 0.07 | 0.634 | 0.30 | 0.046 |
| miR-103a | -0.03 | 0.844 | -0.04 | 0.789 | -0.03 | 0.680 |

Data are reported as Spearman rank correlation (*r*). Abbreviations: adipose tissue insulin resistance (AT-IR); free fatty acids (FFAs); Ra, rate of appearance (Ra).

**TABLE S5** Correlations between circulating cytokines and adipose tissue insulin resistance (AT-IR).

| **Variables** | ***r*** | ***p* value** |
| --- | --- | --- |
| sCD163, ng/mL | 0.36 | 0.027 |
| TNFα, pg/mL | 0.37 | 0.022 |
| IL-1β, pg/mL | 0.35 | 0.039 |
| IL-6, pg/mL | 0.32 | 0.048 |
| IL-10 pg/mL | 0.27 | 0.101 |

Data are reported as Spearman rank correlation (*r*). Abbreviations. Interleukin (IL), soluble cluster of differentiation 163 (sCD163), tumor necrosis factor alpha (TNFα).

**FIGURE S1** Representative graph of nanoparticle tracking analysis of EVs.


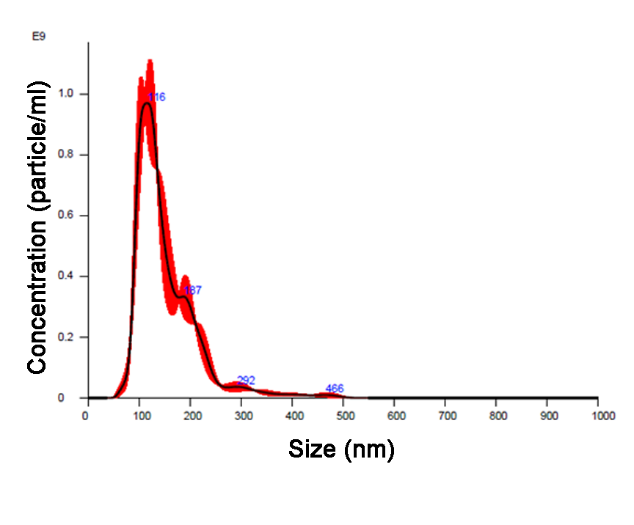


**FIGURE S2** Serum miRNA expression according to liver steatosis (A-G), hepatocyte ballooning (H-N), and lobular inflammation (O-U). Liver steatosis has been reported as mild (1), moderate (2), and severe (3). Hepatocyte ballooning has been reported as none (0), mild (1), and moderate (2). Lobular inflammation has been reported as absent (0) or present (1), due to the low number of patients with moderate inflammation. Serum miRNAs expression has been reported in absolute count (log_2_). *p* values were calculated by Kruskal-Wallis test (A-N) or Mann-Whitney test (O-U).


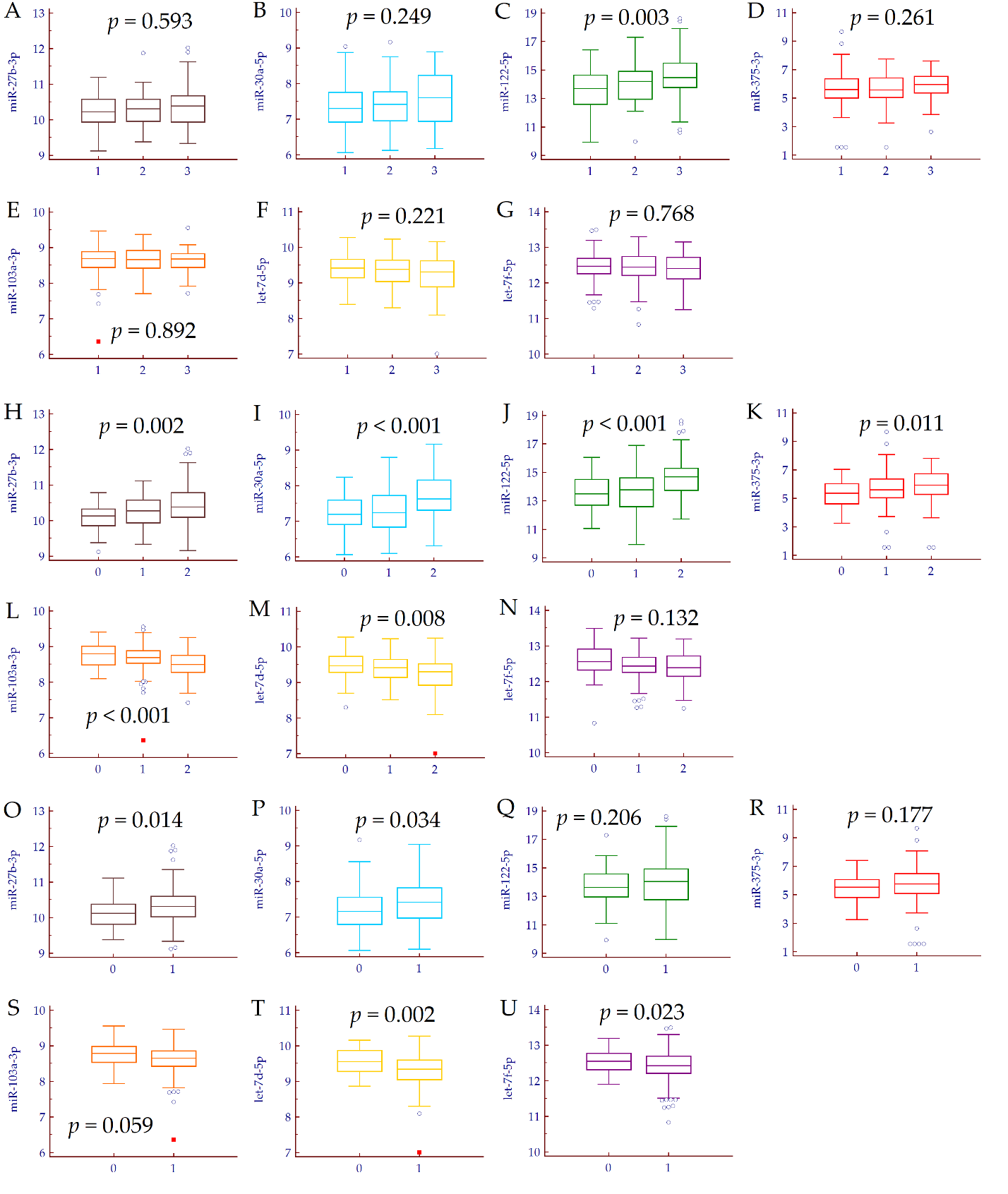


**FIGURE S3** Correlation between miRNAs and glycerol rate of appearance, circulating free fatty acids, and adipose tissue insulin resistance. *p* values were calculated by Spearman’s correlation test. Abbreviations: adipose tissue insulin resistance (AT-IR), free fatty acids (FFA), rate of appearance (Ra).

**
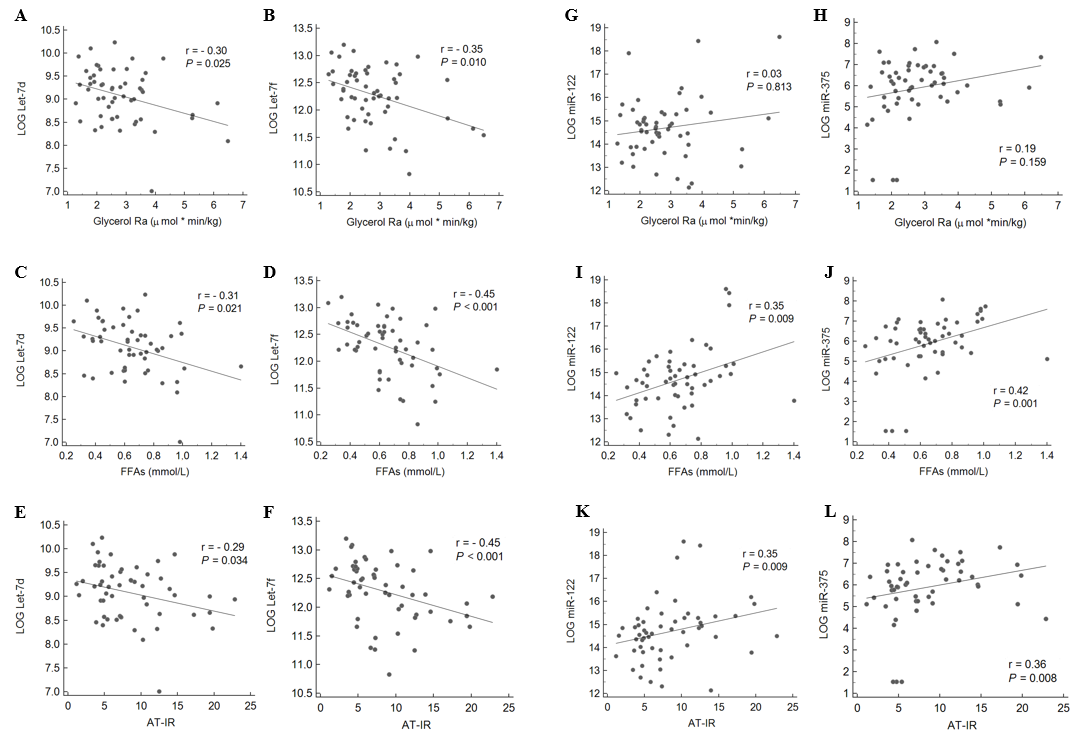
**

**FIGURE S4** Hepatic expression of miR-122-5p (A), miR-375-3p (B), let-7d-5p (C), and let-7f-5p (D) in CDAA-fed mice compared to CSAA-fed controls. *p* values were calculated by Mann-Whitney test. Abbreviations: 24 weeks (24w), choline-deficient L-aminoacid–defined diet (CDAA), choline-sufficient L-amino acid–defined diet (CSAA). *p* values were calculated by Mann-Whitney test.


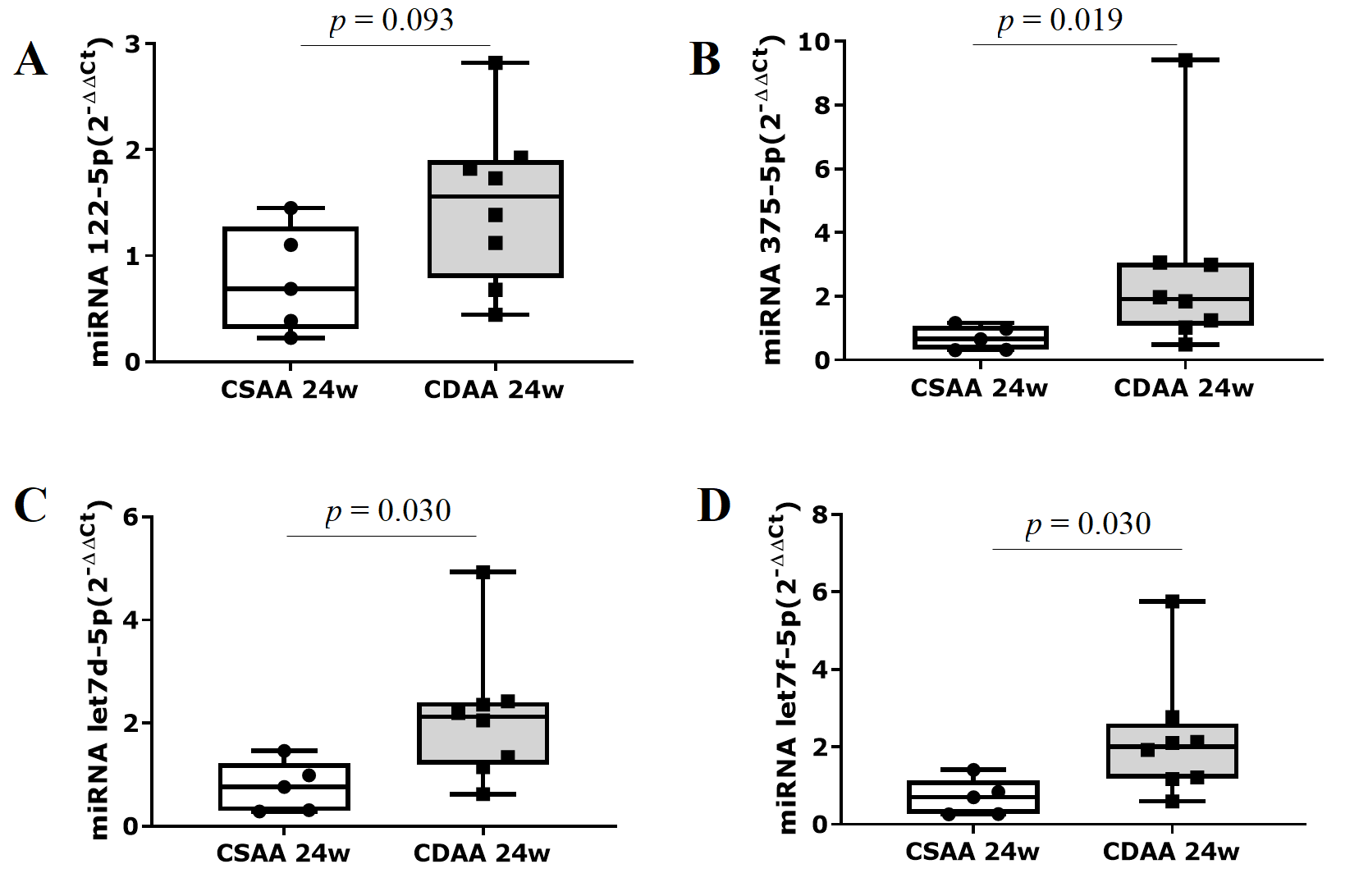

Supplement: Supplementary file 1 — Data S1. [file APT-62-22-s001.doc]
